# Mechanisms of Photoredox Catalysis Featuring Nickel–Bipyridine Complexes

**DOI:** 10.1021/acscatal.4c02036

**Published:** 2024-05-29

**Authors:** David A. Cagan, Daniel Bím, Nathanael P. Kazmierczak, Ryan G. Hadt

**Affiliations:** † Division of Chemistry and Chemical Engineering, Arthur Amos Noyes Laboratory of Chemical Physics, California Institute of Technology, Pasadena, California 91125, United States; ‡ Institute of Organic Chemistry and Biochemistry, The Czech Academy of Sciences, Flemingovo nám. 2, Prague 6 166 10, Czech Republic

**Keywords:** cross-coupling reactions, metallaphotoredox catalysis, organic transformations, photoredox catalysis, mechanism, electronic structure

## Abstract

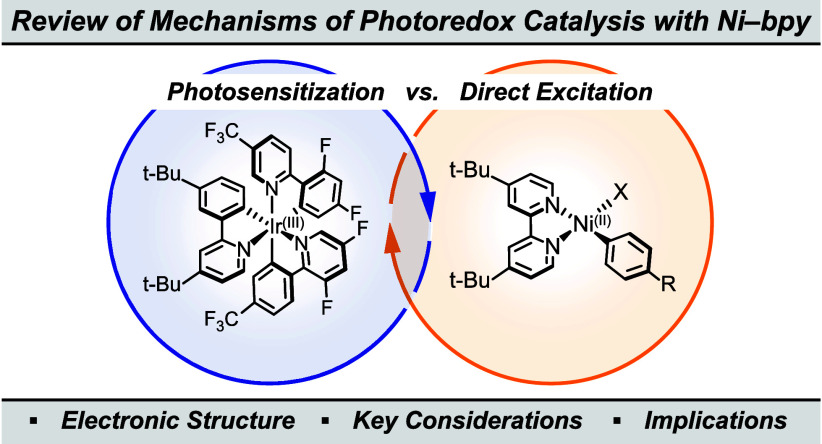

Metallaphotoredox catalysis can unlock useful pathways for transforming organic reactants into desirable products, largely due to the conversion of photon energy into chemical potential to drive redox and bond transformation processes. Despite the importance of these processes for cross-coupling reactions and other transformations, their mechanistic details are only superficially understood. In this review, we have provided a detailed summary of various photoredox mechanisms that have been proposed to date for Ni–bipyridine (bpy) complexes, focusing separately on photosensitized and direct excitation reaction processes. By highlighting multiple bond transformation pathways and key findings, we depict how photoredox reaction mechanisms, which ultimately define substrate scope, are themselves defined by the ground- and excited-state geometric and electronic structures of key Ni-based intermediates. We further identify knowledge gaps to motivate future mechanistic studies and the development of synergistic research approaches spanning the physical, organic, and inorganic chemistry communities.

## 1 Introduction

Pd-catalyzed cross-coupling reactions have transformed organic chemistry with their synthetic contributions to drug discovery and development.^1−3^ Although subtle differences emerge between reactions, the majority of Pd-catalyzed couplings leverage a mechanism featuring dominantly two-electron processes: oxidative addition, transmetalation, and reductive elimination.^4^ Going beyond Pd, a precious metal and limited resource, significant strides have been made toward more sustainable approaches to catalysis. These advances feature critical contributions from methodology-driven research into homogeneous cross-coupling catalysis by first-row transition metal complexes, which are becoming more widely adopted for enabling the construction of new C–X (X = C, O, N, F, etc.) bonds.^5−7^ Mechanistic studies highlight the complexities of these ground-state cross-coupling reactions, but also bring to light new possibilities stemming from one-electron redox processes and the variety of intermediates involved in the underlying bond-formation and bond-rupture processes.^8^

**Figure 1:**
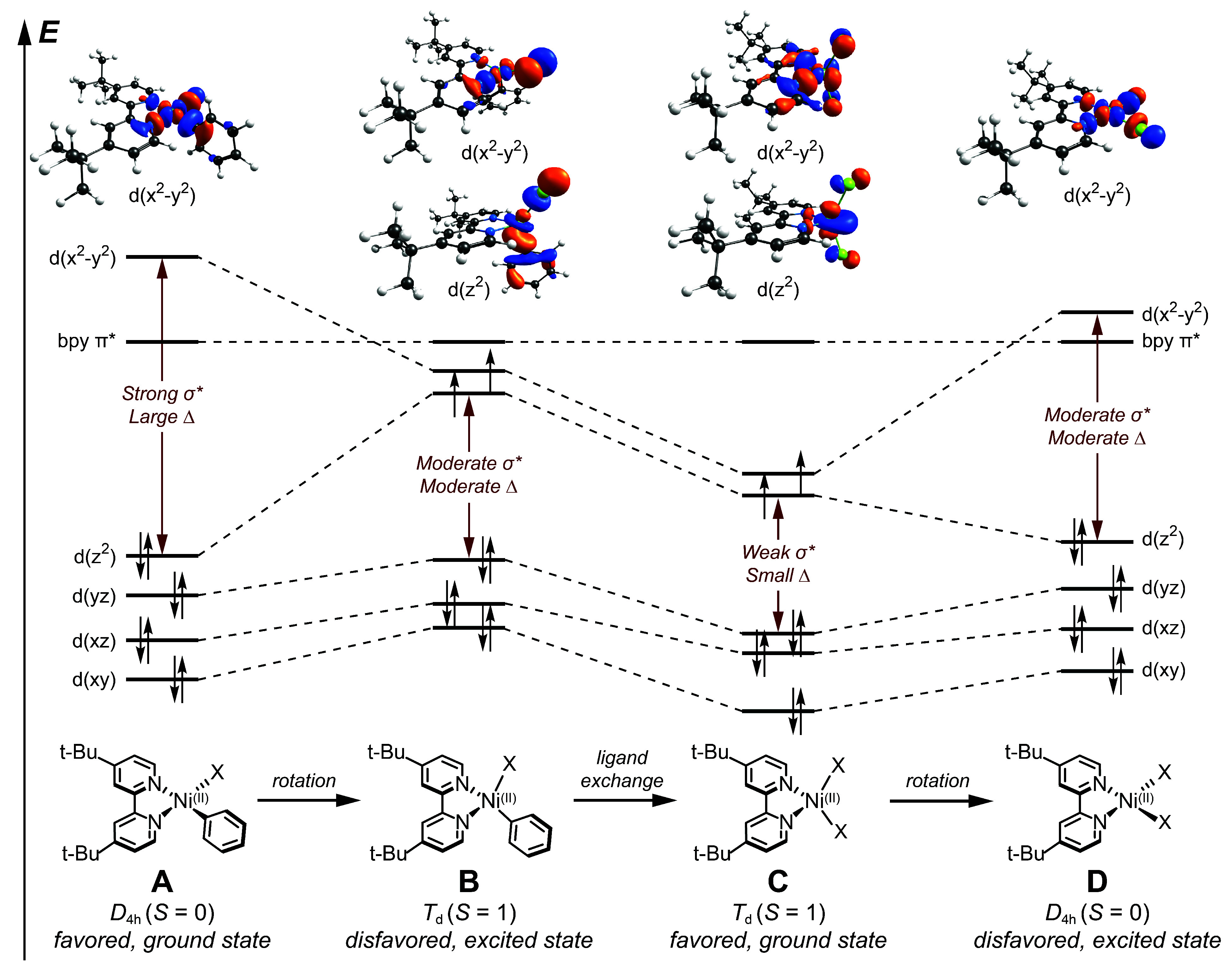
Qualitative molecular orbital correlation diagram of four Ni(II)–bpy species of potential relevance in photocatalytic pathways; each feature distinct geometric and electronic structures, ligand field splitting energies (Δ), and σ* effects. The Ni(II)–bpy aryl halide (A) adopts a square planar (*D*_4h_) geometry, leading to a diamagnetic *S* = 0 ground state. The high spin *S* = 1 geometry (B) is observed as a relaxed excited-state intermediate; population of the 3*d*(x^2^–y^2^) orbital induces a rotation into a pseudo-*T*_d_ geometry. Ni(II)–bpy dihalide (C) is stable as a *T*_d_ triplet ground state. For completeness, we also show this complex in a square planar geometry (D). This singlet state is energetically disfavored and yet to be identified to date. Select molecular orbitals (computed with DFT at the B3LYP/def2-TZVP^48−50^ level of theory) are depicted at the top of the figure for illustration of σ* effects.

Ni-mediated catalysis has emerged as a key alternative to Pd, as it can access a range of formal oxidation and/or spin states (Figure 1) and facilitate numerous complex substrate transformations.^9−11^ In addition to metal redox, ligand-based redox (i.e., ligand noninnocence and potential multireference character)^12−15^ further increases reaction complexity by providing important, yet poorly understood, electronic structure contributions. These can result in noble-metal-like reactivity in base-metal catalysts and provide a basis for transformative structure/function relationships.^7^

Metallaphotoredox catalysis has had a profound influence on many areas of organic chemistry, including cross-coupling reactions. This approach uses photosensitizers to generate metal-based intermediates that can be active in dark cycles.^16−22^ These intermediates often form due to their propensity for single electron transfer (SET).^23^ Photosensitizers can additionally transfer energy to metal complexes to form reactive excited states.^24,25^ The merger of photoredox catalysis with Ni–bipyridine (bpy) complexes has claimed a prominent place in the organic, inorganic, and physical chemistry communities owing to its wide synthetic utility and rich photophysical aspects.^20,26−32^ In addition to light absorption by the photosensitizers present in reaction mixtures (often cyclometalated Ir(III) heteroleptic complexes^33−35^), these Ni–bpy cocatalysts also absorb strongly across the UV–vis region and can directly harvest light to access key excited states.^22,36−40^ In principle, ultrafast spectroscopic methods should be critical to studying the photophysical processes that undergird the overall chemical bond transformations.^41^ However, as discussed below, there are often strongly competing intramolecular excited-state relaxation pathways, and care needs to be taken to account for low quantum yield processes that can be difficult to probe directly using time-resolved spectral methods. Overall, the elucidation of mechanistic routes requires the knowledge of both light- and thermally driven components and the interplay between them. As discussed below, this has proven to be a difficult task for light-driven, Ni-mediated catalytic cycles, and our overall understanding of how photon energy drives organic transformations is still superficial.

The aforementioned progress motivates further efforts to elucidate the geometric and electronic structures of critical inorganic species and photoinduced states that are involved in metallaphotoredox cross-coupling reactions. We believe these knowledge gaps can be addressed by a synergistic combination of synthesis, spectroscopy, and computation to define electronic structure contributions to reactivity, and we hold that there is significant general potential linked to leveraging these complexities for cross-coupling catalysis. To do so, however, significant strides need to be made toward detailed and fundamental studies of discrete light and dark reaction steps that constitute photoredox catalytic cycles. Ultimately, in concert with additional methodological studies, this understanding will help inform chemists how to leverage the inherent properties of first-row transition metals and, thus, guide academic and industrial research toward sustainable approaches for bond constructions in organic synthesis.

While previous reviews have highlighted the tremendous advancements made in the development of new photoredox-enabled transformations,^19,20,31^ this review seeks to compare and evaluate mechanisms that have been proposed in the literature, with a focus on Ni–bpy complexes. We note that additives can influence the catalytic pathway. However, mechanistic analysis of their contributions is quite limited. Thus, while potentially important to consider, this review does not provide a complete picture of their potential mechanistic roles. Given the growing importance of ground- and excited-state processes in metallaphotoredox catalysis, the review first features a brief electronic structure primer, which discusses key aspects of different electronic states of Ni at a broadly accessible level. We subsequently provide a summary and comparison of proposed photoredox mechanisms. Divided into two main sections, we first summarize mechanisms featuring key photosensitization steps. Secondly, we discuss mechanisms that feature direct excitation of Ni-based species for bond-homolysis-driven dark cycle initiation or excited-state bond formation reactions. The mechanistic summaries are further bolstered by “Key Consideration” sections designed to highlight the importance of Ni-based intermediates and their electronic structures. By doing so, we hope to 1) demonstrate the importance and need for further mechanistic studies of metallaphotoredox reactions, even beyond Ni, and 2) highlight the interdisciplinary nature of this growing area, hopefully motivating future synergistic contributions that will span the physical, organic, and inorganic chemistry communities.

## 2 Nickel Electronic Structure Primer

Prior to embarking on our review of light-activated catalytic cycles featuring Ni complexes, it is valuable to consider the distinct electronic structures of the commonly invoked Ni intermediates. Even within a given oxidation state, such as Ni(II), disparate geometries, spin states, and ligand field strengths can lead to unique properties for different species.^22^ These changes have direct implications for evaluating the plausibility of ground- and excited-state reactivity, including mechanistic steps such as light harvesting, energy/electron transfer, and electrophile activation.

Nickel is most stable in the 2+ oxidation state with a *d*^8^ electron configuration.^42^ Many stable four-coordinate Ni(II) species are known, and several feature prominent roles in the mechanisms outlined below. Given the importance of these species and their reactivity, we consider their geometric and electronic structures at length. As this review focuses on Ni–bpy complexes, we assume that two of the four coordination sites are occupied by the bpy ligand. Charge balance requires the remaining two ligands be anionic. Common options in the context of cross-coupling include aryl and halide ligands, which could potentially be arranged in either a square planar (*D*_4h_) or a pseudotetrahedral (*T*_d_) geometry (Figure 1). These options are not independent of the ligand character, as described below.

In the square planar geometry, the vast majority of the σ* character is concentrated in the 3*d*(x^2^–y^2^) orbital, resulting in a large ligand field splitting energy, Δ (Figure 1).^43^ This splitting is greater than the electron–electron repulsion (i.e., spin pairing energy) incurred by having the seventh and eighth electrons occupying the same orbital (the 3*d*(z^2^) orbital here). Thus, it is more energetically favorable to adopt a low-spin, *S* = 0 configuration with a doubly unoccupied 3*d*(x^2^–y^2^) orbital. If the ligands are rotated into a pseudo-*T*_d_ geometry, multiple nearly degenerate orbitals share the σ* character, leading to a small Δ relative to the square planar case and a high-spin, *S* = 1 *d*^8^ configuration. (Note that calculations of molecular orbital energies for related pseudo-*T*_d_ Ni(II) complexes suggest two main σ* orbitals, as opposed to the three σ*-orbitals found in a perfect tetrahedron.)^44,45^ Accordingly, population of the strongly antibonding 3*d*(x^2^–y^2^) orbital in the *D*_4h_ geometry (such as through metal-centered photoexcitation) induces a geometric rotation to the pseudo-*T*_d_ geometry to minimize the σ* overlap.

The choice between a square planar and pseudo-*T*_d_ geometry can thus be understood as a competition between electron repulsion (spin pairing energy) and the ligand field splitting energy.^46^ The *D*_4h_*S* = 0 state pays the energetic penalty for pairing electrons, but it avoids populating the high-lying 3*d*(x^2^–y^2^) orbital and is therefore unaffected by larger values of Δ (Figure 1, **A** and **D**). On the other hand, the pseudo-*T*_d_*S* = 1 state avoids the energetic penalty for pairing electrons in the same orbital, yet it populates both the 3*d*(z^2^) and 3*d*(x^2^–y^2^) orbitals, which each experience an energetic disadvantage according to the magnitude of Δ (Figure 1, **B** and **C**). Accordingly, strong-field ligands favor the square planar geometry, while weak-field ligands favor the pseudo-*T*_d_ geometry.^47^

Herein arises the essential difference between aryl and halide ligands. From the perspective of ligand field theory, the aryl is considered a strong-field ligand, while halides are weak-field ligands.^51^ As such, the gap between the σ* orbital(s) and the remaining, lower-lying 3*d*-orbitals will be large for a Ni(II)–bpy aryl halide species, but comparatively small for Ni(II)–bpy dihalides. For this reason, Ni(II)–bpy aryl halides feature singlet, square planar ground states, while Ni(II)–bpy dihalides feature pseudo-*T*_d_ triplet ground states. Note that pseudohalide ligands (such as alkoxides or acetates) result in similar electronic structures as halides; alkyl ligands behave as aryls, but with larger values for Δ, as they are stronger σ-donors.

For Ni(II)–bpy aryl halides vs Ni(II)–bpy dihalides, their distinct geometries and spin states have significant implications for electron transfer in catalysis owing to the divergent energies of the redox-active molecular orbital (RAMO). For the ground-state Ni(II)–bpy aryl halide, the lowest unoccupied molecular orbital (LUMO) is not metal-based. The strong σ* overlap of 3*d*(x^2^–y^2^) orbital raises its energy above the bpy π* orbital manifold. As such, the first reduction event for Ni(II)–bpy aryl halide is observed on the bpy ligand rather than the metal.^52,54^ Reduction of the complex results in an anionic [Ni(II)–bpy^•–^ aryl halide]^−^, which slowly decomposes to a three-coordinate Ni(I)–bpy aryl species.^52,53^ When the aryl ligand is replaced by a halide, the reduction in σ-donation strength and associated antibonding character leads to a significant decrease in the 3*d*(x^2^–y^2^) orbital energy. Furthermore, as the ground-state Ni(II)–bpy dihalide adopts a pseudo-*T*_d_ geometry, an additional stabilization in the Ni-based RAMO is expected. One-electron reduction of this complex affords a doubly occupied σ* 3*d*(z^2^) orbital, resulting in ejection of a halide to give a Ni(I)–bpy halide complex.^55,56^

The reduction potential of the complex trends with the energy of the LUMO. In Ni(II)(^*t*-Bu^bpy)(*o*-tolyl)Cl, the first reduction event is found to be −1.6 V vs SCE, corresponding to electrochemically reversible bpy reduction. Irreversible Ni-based reduction appears at ∼ −1.8 V vs SCE.^57^ By contrast, the first reduction event for Ni(II)(^*t*-Bu^bpy)Cl_2_ is at −1.3 V vs SCE (Ni-based and irreversible).^55,56,58^ The activity of a Ni(II) complex toward reductive steps in a catalytic cycle is dramatically influenced by ligand field strength and coordination geometry;^54,59,60^ similar considerations were also demonstrated for *S* = 1 chiral enantioselective Ni(II)–diimine dihalide cross-coupling catalysts.^44,61,62^ Ligand field analysis of molecular orbital energies indicates the relative plausibility of various catalytic reduction events.

**Figure 2:**
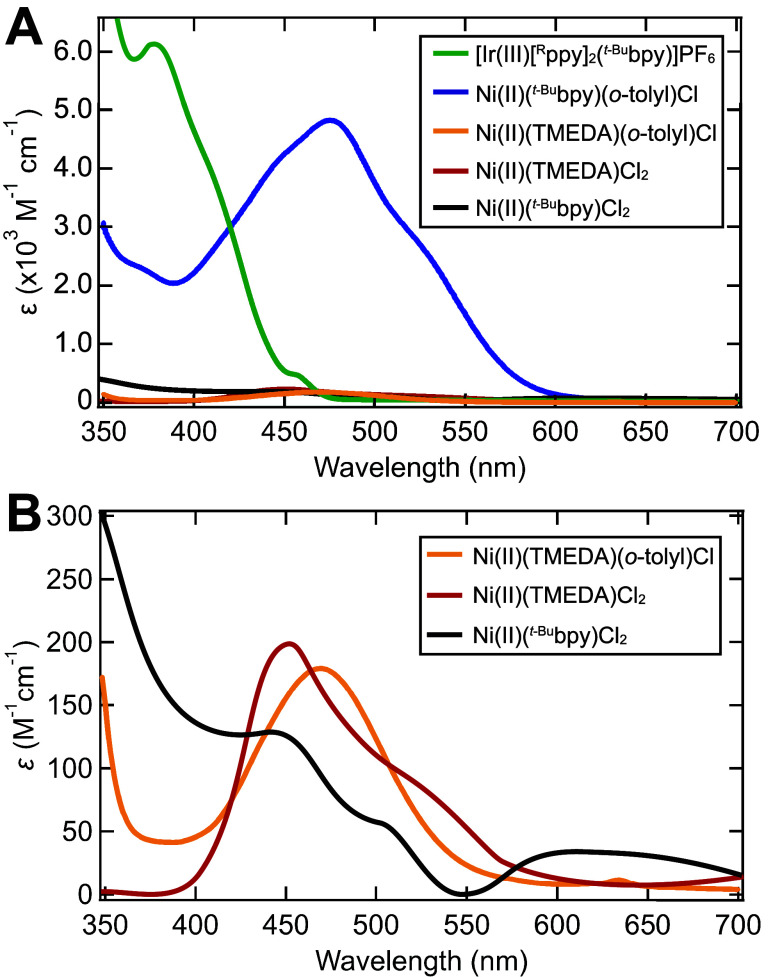
UV–vis absorption spectra of a common Ir(III) photosensitizer and various Ni complexes. (A) Strongly absorbing complexes with charge transfer bands, [Ir(III)[^R^ppy]_2_(^*t*-Bu^bpy)]PF_6_ (green line, R = 2-(2,4-difluorophenyl)-5-trifluoromethyl), and Ni(II)(^*t*-Bu^bpy)(*o*-tolyl)Cl (*S* = 0, blue line), are highlighted. (B) An expanded view of complexes with only ligand field transitions in the visible region, Ni(II)(TMEDA)(*o*-tolyl)Cl (*S* = 0, orange line), Ni(II)(TMEDA)Cl_2_ (*S* = 1, red line), and Ni(II)(^*t*-Bu^bpy)Cl_2_ (*S* = 1, black line). Solvent = THF. Spectra were digitized and scaled with permission from references (36), (Copyright 2018 American Chemical Society) (38) (Copyright 2022 American Chemical Society), (65) (available under a CC-BY NC 3.0 Deed license, copyright 2024 Bryden and Zysman-Colman), (66) (Copyright 2016 John Wiley and Sons) and (67) (Copyright 2020 American Chemical Society).

In addition to redox potentials, the geometric and electronic structures of Ni–bpy complexes determine their light harvesting ability through the molar absorption coefficients of the UV–vis transitions. As a ligand with a significant π-conjugation, the bpy possesses low-lying π*-orbitals capable of backbonding with the metal center. These bpy orbitals serve as acceptors for metal-to-ligand charge transfer (MLCT) transitions in the visible absorption spectrum and possess significant electron delocalization, leading to a large transition dipole moment. Replacement of the bpy ligand for aliphatic *N,N,N′,N′*-tetramethylethylenediamine (TMEDA) exemplifies this point, where only ligand field bands become possible, leading to reduced values of ε (Figure 2).^38,63,64,66^ Ni(II)–bpy aryl halide complexes exhibit MLCT transitions (350 nm−550 nm) that possess molar extinction coefficients of comparable magnitude as iridium photosensitizers (ε = 10^3^ – 10^4^ M^–1^ cm^–1^),^36,38,65,67^ rendering these Ni(II) species competitive for photocatalytic light harvesting. Ni(II)–bpy dihalide complexes show orbitally forbidden ligand field transitions in the visible to near-infrared region with ε = 10^1^ – 10^2^ M^–1^ cm^–1^ (Figure 2).^36^

Similar analyses may be conducted for other oxidation states. Three-coordinate Ni(I) complexes adopt an approximately planar geometry; while the 3*d*(x^2^–y^2^) σ* interaction is somewhat lessened due to the loss of 4-fold symmetry and consequent orbital overlap, there nonetheless remains a large energetic separation between the 3*d*(x^2^–y^2^) orbital and the remainder of the 3*d*-manifold due to σ* interactions with the bpy and π* interactions with the halide. The *d*^9^ Ni(I) configuration implies single occupation of the high-energy σ*-orbital; however, this is tolerated, and such Ni(I) compounds have been characterized.^54,56,68^ However, further reduction of Ni(I) to Ni(0) requires the introduction of an additional electron into the destabilized σ* 3*d*(x^2^–y^2^) orbital. The reduction potentials for such an event are thought to be high, and it is unclear whether Ni(0) is catalytically accessible^69,70^ (see Reductive SET mechanism below). Indeed, Ni(0)–bpy cyclooctadiene (COD) exhibits a large degree of bpy ligand redox noninnocence and is proposed to exist as Ni(I)(bpy^•–^)(COD).^71^ Interestingly, Ni(I)–bpy halide complexes exhibit MLCT transitions across a wide wavelength range (350 nm −1400 nm) and have molar extinction coefficients of equal or greater magnitude than Ni(II)–bpy aryl halides, marking yet another competitive light-harvesting species in photocatalytic cycles.^56^

## 3 Summary and Comparisons of Proposed Photoredox Mechanisms

Key consideration sections are provided for each of the mechanisms summarized herein, with the goal of connecting these considerations to experimental observations that are emphasized across all Ni–bpy-based photoredox mechanisms, both in terms of direct excitation and photosensitization.

### 3.1 Photosensitization

#### 3.1.1 Reductive SET

**Figure 3:**
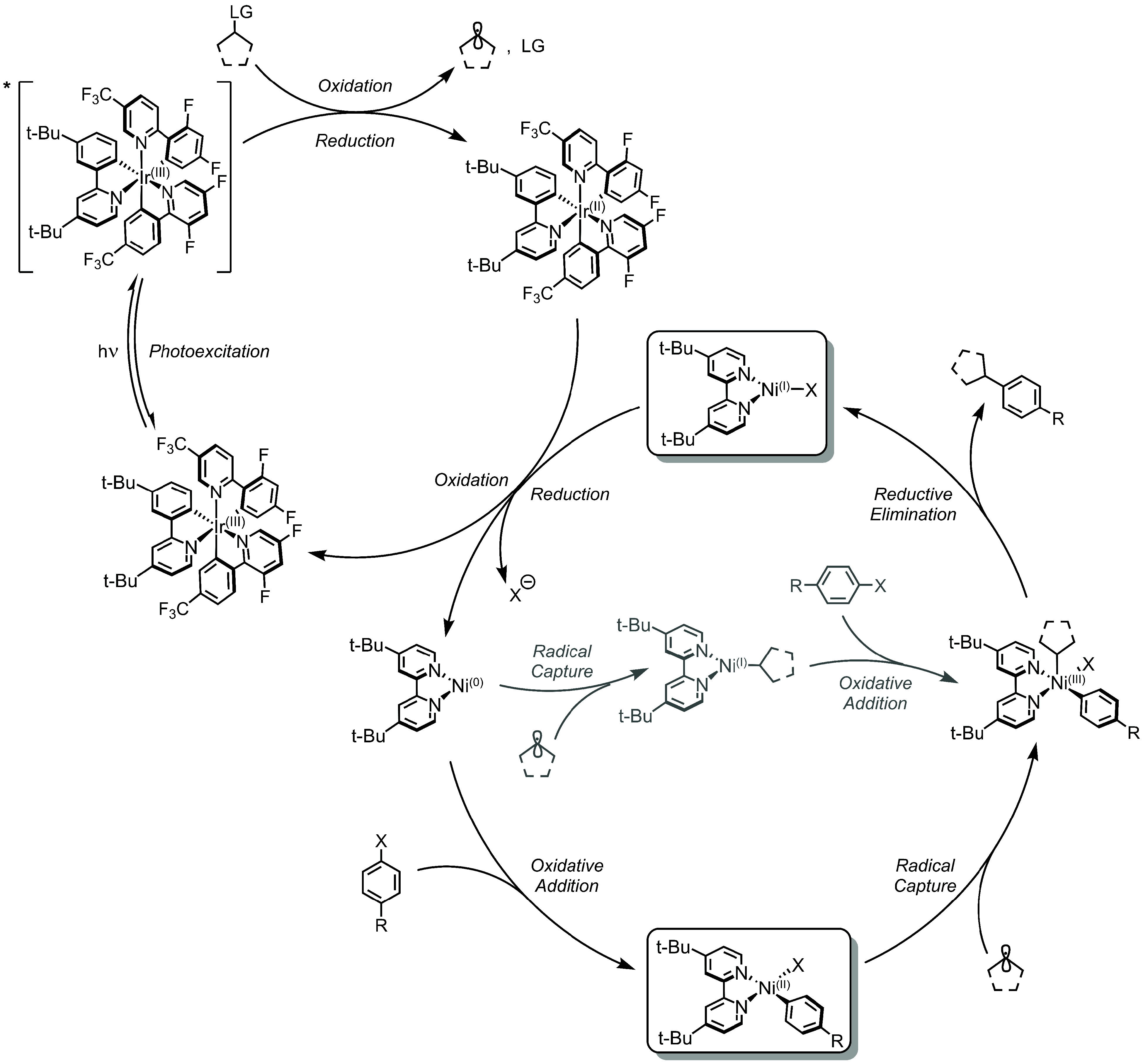
Proposed Reductive SET mechanism. C(sp^2^)–C(sp^3^) coupling is presented as a representative example. LG = leaving group.

The first metallaphotoredox reactions using light-activated nickel were reported independently in 2014 by the groups of Molander^29^ and Doyle and MacMillan,^30^ where C(sp^2^)–C(sp^3^) cross-couplings were discovered in reactions combining Ni(0)–bpy, an Ir(III) photosensitizer, and organic coupling partners. The reaction scope was further extended to C(sp^2^)–C(sp^2^) and C(sp^3^)–C(sp^3^) couplings in 2015 and 2016, respectively,^72−74^ then for the activation of aliphatic C–H bonds in 2018,^75^ and to alkyl chloride substrates in 2019^76^ and 2020;^77^ enantioselective cross-coupling was seen a year later.^78^ Based on a thermodynamic redox potential argument, it was speculated that the iridium excited state, *Ir(III), carried out two separate SET events. This mechanism is termed “Reductive SET” herein, as the first (and only) proposed interaction between iridium and nickel is a reduction of Ni(I) to Ni(0) (Figure 3).

In the Reductive SET mechanism, the Ir(III) photosensitizer is the sole excited-state active species. In one SET, *Ir(III) oxidizes the alkyl coupling partner, affording C(alkyl)^•^ and Ir(II). In another SET, Ir(II) reduces a Ni(I)–bpy halide complex (top box, Figure 3) to Ni(0)–bpy, which can undergo oxidative addition with an aryl halide to generate a square-planar (*S* = 0) Ni(II)–bpy aryl halide complex (bottom box, Figure 3). This Ni(II) complex captures the *Ir(III)-generated alkyl radical, and the resultant pentacoordinate Ni(III) species undergoes reductive elimination to form a Ni(I)–bpy halide and the C(sp^2^)–C(sp^3^) cross-coupled product. The cycle continues upon further reduction of Ni(I)–bpy halide by Ir(II) to Ni(0)–bpy and Ir(III).

#### 3.1.2 Key Considerations for the Reductive SET Mechanism

##### 3.1.2.1 Ir(III) Acts as the Sole Light-Harvesting Species

This is a critical point for any photoredox cycle featuring multiple intermediates that could absorb photons with energies matching those of the irradiation source. For example, Ni(II)–bpy aryl halide complexes (bottom box, Figure 3) are now known to be photoactive in C(sp^2^)–C(sp^3^) cross-coupling upon direct excitation via a Ni(II)–C(aryl) to Ni(I) + C(aryl)^•^ bond homolysis step.^36−38,79^ Even a small amount of photogenerated Ni(I) through this alternative step may be sufficient to catalyze the reaction. These examples are discussed in Section 3.1.9. Importantly, both the Ir photosensitizer and the Ni(II)–bpy aryl halide complexes absorb light in the visible region with molar extinction coefficients of 10^3^ M^–1^ cm^–1^ (Figure 2). The molar absorptivities of the various Ni intermediates possible in the reaction cycle are largely unknown.

##### 3.1.2.2 *Ir(III) Is Sufficiently Oxidizing to React with Alkyl Substrates, Doing so Preferentially

Redox interactions between *Ir(III) and substrate can be probed through electrochemical measurements and the oxidation state of the Ir complex tracked by absorption spectroscopy. Interactions between *Ir(III) and species in solution other than the organic substate, including any Ni complexes in the putative cycle, are possible and should be evaluated. For example, the alkyl substrates used in the above-mentioned work have accessible oxidation potentials of ∼1 V versus SCE,^29,30,80^ but these neighbor the oxidation potential of Ni(II)–bpy aryl halide (∼0.8–0.9 V versus SCE). As will be seen below, related interactions between *Ir(III) and Ni complexes are invoked in the Oxidative SET mechanism (Section 3.1.3). Furthermore, both SET and triplet energy transfer (^3^EnT) are possible from *Ir(III) to Ni(II),^81^ further complicating analyses (see Sections 3.1.3 and 3.1.7).

##### 3.1.2.3 Ni(0)–bpy Undergoes Oxidative Addition, While Ni(I)–bpy Halide Does Not

Both Ni(0) and Ni(I) can undergo oxidative addition with aryl halides. However, Ni(I)–Ni(III) oxidative addition would divert the proposed Reductive SET mechanism from Ni(0)–Ni(II) oxidative addition. The reactivity of Ni(0) and Ni(II) vs Ni(I) and Ni(III) are distinct. Furthermore, the presence of Ni(I) and Ni(III) can lead to facile comproportionation to *S* = 0 Ni(II)–bpy aryl halide and *S* = 1 Ni(II)–bpy dihalide,^82^ another chemically distinct species that is not considered in this mechanism but is important for others (Section 3.1.9). Additionally, Oderinde, Johannes, and co-workers noted the reduction potential of Ir(II) is scarcely able to reduce various Ni(I) complexes, finding their potentials to be similar ([Ir^III^/Ir^II^] = −1.37 V vs SCE, [Ni^I^/Ni^0^] = −1.41 V vs SCE), and that Ni(0) is ineffective to turn over the cycle.^69^ Further disfavoring Ni(0), Gutierrez, Martin and co-workers found that Ni(II)–bpy dihalide complexes engage in rapid, facile comproportionation with Ni(0)–bpy species in solution, affording Ni(I)–bpy halide species.^83^ However, Plasson, Fensterbank, Grimaud and co-workers argued that Ni(0) is indeed a vital source of Ni(II)–bpy aryl halide,^84^ and Bahamonde and co-workers argued that oxidative addition to Ni(0) outcompeted the comproportionation reaction, supporting an Oxidative SET mechanism, though ^3^EnT pathways were not discarded^85^ (see Section 3.1.7). Altogether, the requirement of Ni(0) for catalytic cycle turnover is still debated.

##### 3.1.2.4 Alkyl Radicals Are Preferentially Captured by Ni(II), Not Ni(0)

Given that Ni(0) and Ni(II) complexes are present in the proposed mechanism, a comparison between the relative rates of radical capture by both of these species would help confirm the Ni(II) to Ni(III)–alkyl hypothesis. Computations by Molander, Kozlowski, and co-workers suggest both oxidation states should be productive toward radical capture.^86^ While kinetic analysis for radical capture at Ni(II) was recently reported (*k* = 10^6^ – 10^7^ M^–1^ s^–1^),^87^ we are unaware of studies for C(alkyl)^•^ capture by Ni(0).

##### 3.1.2.5 Ir(II) Is Sufficiently Reducing to Regenerate Ni(0) and Ir(III)

The presence of Ir(II) presupposes that Reductive SET is indeed operative (see point 2 above). Given the highly reducing nature of Ir(II), one must also consider its potential interaction with Ni(II) and Ni(III). Reduction of Ni(II) to Ni(I) would present an alternative mechanistic route, potentially favoring a Ni(I/III) catalytic cycle (see point 3). Additionally, Neurock, Minteer, Baran, and co-workers reported that pentacoordinate Ni(III) complexes are readily reduced to Ni(II) via Ni–X heterolysis.^55^ It is possible the Ni(III) species could be intercepted by Ir(II) prior to reductive elimination and thereby be diverted from the cycle making C(sp^2^)–C(sp^3^) bonds. Again, relative reactivity rates between Ir(II) and the relevant Ni species would prove invaluable for mechanistic insight.

There have been limited experimental mechanistic studies conducted on this reaction, but one notable example is the work by Lloyd-Jones and co-workers in 2022.^88^ Careful kinetic analysis using radiolabeled substrates and ^13^C NMR identified the Ni(II)–bpy aryl halide as a genuine intermediate. From the kinetic modeling, three plausible mechanisms were proposed for the reaction, including one which is akin to the Reductive SET mechanism illustrated above. Interestingly, this mechanistic possibility was the only one of the three the researchers were able to rule out. The remaining two mechanisms proposed by Lloyd-Jones and co-workers centered around *Ir(III) promoting a photoinduced Ni–halide bond homolysis step, referred to here as “Photosensitization for Homolysis” (see Section 3.1.5). However, the three mechanisms considered therein are not an exhaustive list, as noted by the authors.^88^*Nonetheless, based on these considerations and the recent kinetics study, the initially proposed Reductive SET mechanism is unlikely operative.* Additional detailed experimental studies are necessary, however, particularly addressing the five points outlined above.

#### 3.1.3 Oxidative SET

**Figure 4:**
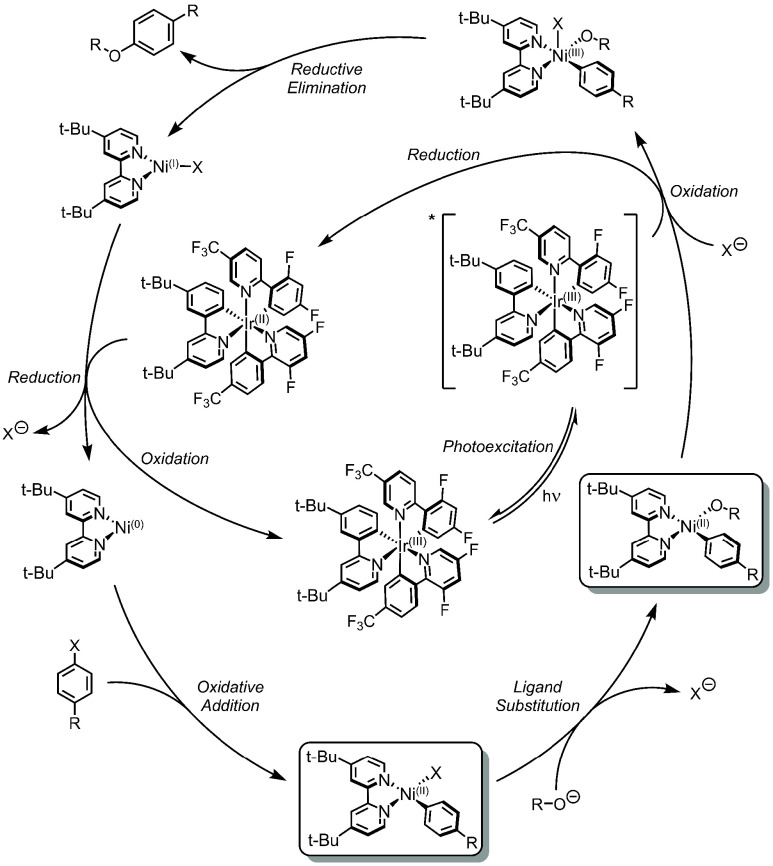
Proposed Oxidative SET mechanism. C(sp^2^)–O coupling (alcohols) is shown as a representative example.

The expansion of dual Ni/Ir metallaphotoredox reactions to C(sp^2^)–X coupling led to an additional mechanistic hypothesis, Oxidative SET, as proposed for C(sp^2^)–N coupling by Jamison and co-workers in 2015^89^ and C(sp^2^)–O/N coupling by MacMillan and Buchwald and co-workers^28,90^ in 2015 and 2016, respectively. In the Reductive SET mechanism for C–C bond coupling, Ir(II) interacted with a Ni(I)–bpy halide complex, reducing it by one electron in a dark reaction. Keeping with the naming convention adopted herein, the Oxidative SET mechanism features a SET wherein *Ir(III) oxidizes a Ni(II)–bpy aryl alkoxide complex (right box, Figure 4), leading to a Ni(III) species and Ir(II). As in Reductive SET, the Ir(III) complex acts as the sole excited-state active species in Oxidative SET. Ir(II) reduces a Ni(I)–bpy halide species to generate Ni(0)–bpy, which undergoes oxidative addition of an aryl halide coupling partner to form a Ni(II)–bpy aryl halide species (bottom box, Figure 4). Ligand substitution of the alcohol (or amine) via the assistance of exogenous base generates the aforementioned four-coordinate, square-planar Ni(II)–bpy aryl alkoxide (right box, Figure 4). The critical chemical impetus behind this mechanism is the Ni(III)-promoted reductive elimination of the C–X product, akin to the one-electron oxidation chemistry developed by Hillhouse and co-workers.^91,92^ Initial reports founded this reaction scheme on the basis of redox potentials and reductive elimination thermodynamics for Ni(II) vs Ni(III).

#### 3.1.4 Key Considerations for the Oxidative SET Mechanism

##### 3.1.4.1 Ni(II)–bpy Aryl Alkoxide Is the SET Partner with *Ir(III)

While oxidation of the Ni(II)–bpy aryl alkoxide species to formal Ni(III) may be necessary to drive reductive elimination, there are additional Ni species present, including the Ni(II)–bpy aryl halide complex. It is currently unclear why *Ir(III) would preferentially oxidize one and not the other. Additionally, if Ir(II) is competent for the reduction of Ni(I) to Ni(0), why either of these Ni(II) species is not also reduced presents an open question. As demonstrated by Diao and co-workers, electrochemical reduction of Ni(II)–bpy aryl halide to Ni(I)–bpy aryl represents an important step in alternative cross-coupling mechanisms.^52^ Indeed, through electrochemical and computational mechanistic analysis, Oderinde and co-workers presented an alternative mechanism wherein Ni(II)–bpy aryl halide is reduced by Ir(II) to form Ni(I)–bpy aryl.^53^ This reduction was also suggested to be important through computations by Molander, Gutierrez, and co-workers.^93^ Thus, there may be additional, alternative routes aiding in or solely responsible for the production of cross-coupled product. Mechanistic analyses of these discrete steps, particularly those involving key interactions between Ir and Ni, are needed.

##### 3.1.4.2 The Proposed Cycle Rests on Ni(0)–bpy/Ni(II)–bpy Aryl Halide as the Starting Source of Nickel

While Oxidative SET features Ni(0)–bpy to Ni(II)–bpy aryl halide oxidative addition, Buchwald, MacMillan, and co-workers also find that beginning with high-spin (*S* = 1) Ni(II)–bpy dichloride is suitable for the transformation.^90^ Indeed, the substrate scope and product yields are all achieved using this NiCl_2_ starting species, not Ni(0). *This switch in Ni precursor presents a dilemma, namely that the electronic structure, redox potential, and behavior of high-spin Ni(II)–bpy dihalide vary considerably compared to the low-spin Ni(II)–bpy aryl halide that arises from Ni(0).* Little to no experimental mechanistic analysis on this reaction beginning with Ni(0) has been reported.^94^

Detailed follow-up work was done on this reaction by Nocera and co-workers^95^ to interrogate the cycle beginning with Ni(II)–bpy dihalide, and their analysis argued against the Oxidative SET mechanism (see SET for Active Ni(I)). Furthermore, in the closely related C(sp^2^)–O cross-coupling of aryl acetate substrates,^96^ a ^3^EnT mechanism was favored over Oxidative SET by experimental mechanistic work.^97^ We therefore find it plausible that either the Oxidative SET mechanism is not operative for C(sp^2^)–X coupling, or it is only operative when beginning with a Ni(0)–bpy/Ni(II)–bpy aryl halide precursor combination–a pathway still underexplored mechanistically. The electronic structure of the Ni precursor is nontrivial for dictating the mechanistic pathway for catalysis (see Section 2).

#### 3.1.5 Photosensitization for Homolysis (SET vs ^3^EnT)

**Figure 5:**
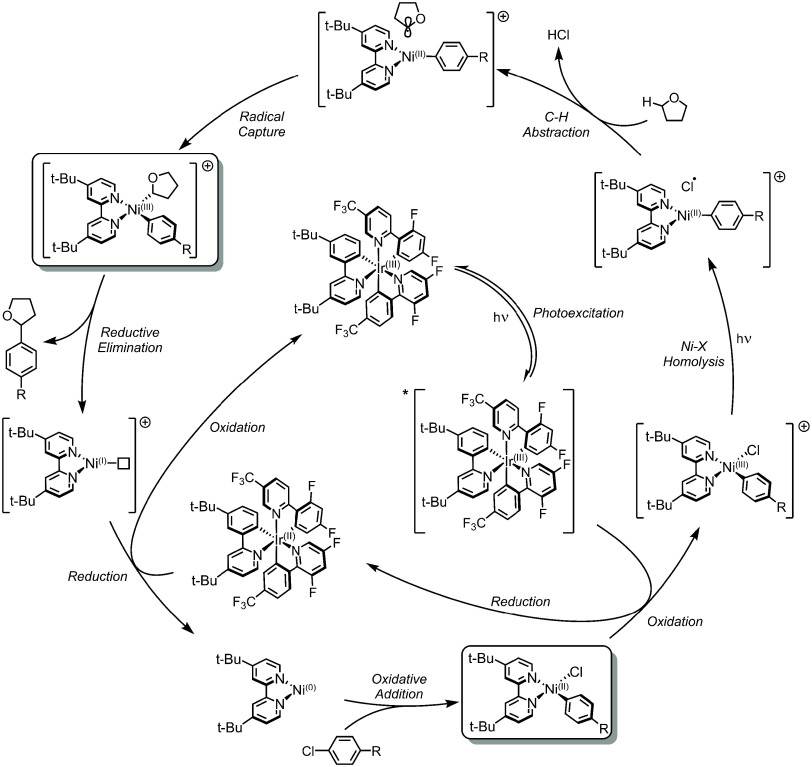
Proposed Photosensitization for Homolysis Mechanism involving SET.^57^

**Figure 6:**
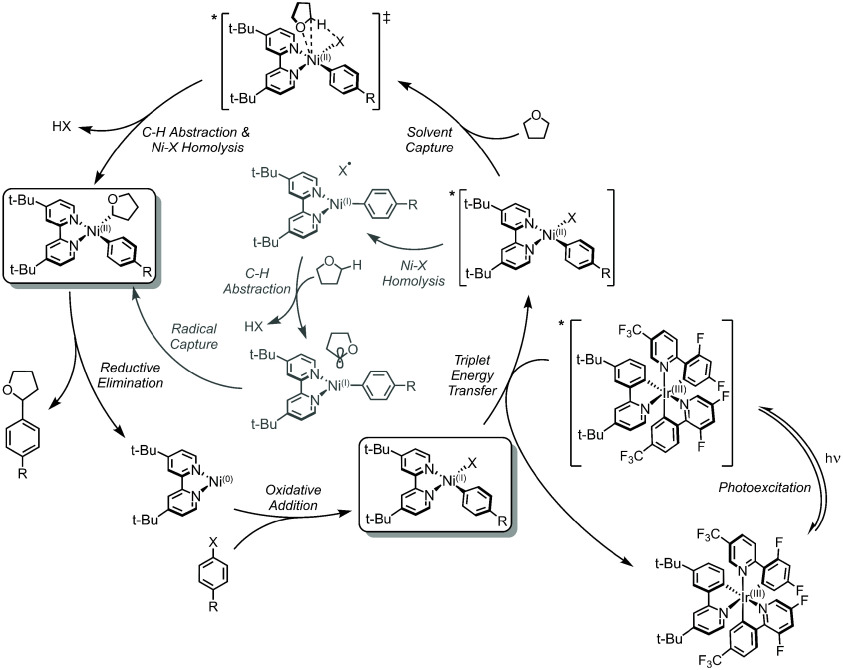
Proposed Photosensitization for Homolysis Mechanism involving ^3^EnT.^98^

Two versions of the Photosensitization for Homolysis mechanism were invoked by concurrent works in 2016, one by Doyle, Shields and co-workers^57^ and another by Molander and co-workers.^98^ The two studies identified a simplified version of a C(sp^2^)–C(sp^3^) coupling reaction that no longer required an easily oxidized alkyl coupling partner for C^•^ generation. Instead, the groups found ethereal solvent (THF) to be a suitable C(sp^3^) source. The two versions of the Photosensitization for Homolysis mechanism are shown below, one involving SET (Figure 5), another ^3^EnT (Figure 6).

The original SET mechanism of Doyle, Shields, and co-workers^57^ involves oxidative addition of an aryl halide coupling partner to Ni(0)–bpy, affording Ni(II)–bpy aryl halide. Rather than engaging an organic substrate, *Ir(III) oxidizes this Ni(II) complex to a four-coordinate, cationic [Ni(III)–bpy aryl halide]^+^ species and Ir(II). Ir(III) is no longer the primary light-absorber in this mechanism. Here the Ni(III) intermediate must undergo photon absorption as well, which promotes halide-to-Ni(III) ligand-to-metal charge transfer (LMCT). This electron excitation populates a Ni(III)–X antibonding orbital, resulting in an excited-state bond homolysis, ejection of an in-cage X^•^, and formation of a three-coordinate Ni(II)–bpy aryl species. Note this step is analogous to the excited-state bond homolysis for isolable Ni(III) trihalide species,^99,100^ wherein the apical Ni(III)–X bond cleaves due to a dissociative LMCT excited-state potential energy surface (PES). The X^•^ abstracts a hydrogen atom from neighboring ethereal solvent (THF in this case), generating an in-cage C^•^ and HCl. The C^•^ is captured by the three-coordinate Ni(II)–bpy aryl species, resulting in the formation of the cationic [Ni(III)–bpy aryl alkyl]^+^ complex (upper left box, Figure 5). Rapid reductive elimination follows, affording C(sp^2^)–C(sp^3^) coupled product and Ni(I)–bpy. Ir(II) reduces this Ni(I) species to Ni(0)–bpy, returning Ir(III).

Molander and co-workers^98^ proposed a related catalytic cycle featuring the same Ni(0) to Ni(II) oxidative addition (bottom of Figure 6). However, instead of undergoing subsequent SET, the Ni(II)–bpy aryl halide acts as a ^3^EnT acceptor from *Ir(III). Thus, in this mechanism, Ir(III) is again the sole light-harvesting species. Upon photosensitization, excited *[Ni(II)–bpy aryl halide] can follow either stepwise out-of-cage or concerted in-cage Ni–X bond homolysis and alkyl solvent capture. The product of either process is a Ni(II)–bpy aryl alkyl species (left box, Figure 6), which undergoes thermal reductive elimination to yield the aryl–alkyl coupled product and Ni(0).

#### 3.1.6 Key Points of the Photosensitization for Homolysis Mechanisms

##### 3.1.6.1 In addition to Ir(III), a Ni–bpy Aryl Halide Species Also Absorbs Light

While the cycle in Figure 6 requires photon absorption by Ir(III), additional mechanistic analysis has found that direct irradiation of the reaction mixture with high-energy light (290–315 nm) without the Ir(III) complex also yields the desired cross-coupled product.^98^ Relatedly, the cycle in Figure 5 necessitates additional photon absorption by a Ni(III) complex in the cycle. As such, identifying 1) the relative absorption cross sections and 2) the resulting quantum yields of ensuing processes for the Ir(III), Ni(II)–bpy aryl halide, and cationic [Ni(III)–bpy aryl halide]^+^ species is imperative for evaluating these potential mechanistic pathways. Furthermore, oxidation of Ni(II)–bpy aryl halide has been demonstrated to rapidly afford the aryl halide substrate.^36^ The proposed intermediacy of a [Ni(III)–bpy aryl halide]^+^ species is therefore critical. In order for this species to avoid reduction by iridium or aryl halide reductive elimination, it must 1) remain stable in room temperature solution long enough to outcompete Ir(III) as a light-harvesting species and 2) have an LMCT within the energy range of the excitation source. The Ir(III) complex has a near unity quantum yield (Φ = 1) for *Ir(III) formation,^34,67,101^ which can react through near-diffusion controlled quenching with Ni(II)–bpy aryl halide (*k*_q_ = 10^9^ M^–1^ s^–1^)^57,95^ to regenerate ground-state Ir(III); both *Ir(III) and Ir(II) are thermodynamically suitable reductants for Ni(III).

##### 3.1.6.2 *Ir(III) Can Undergo SET or ^3^EnT with Ni(II)–bpy Aryl Halides

Determination of whether *Ir(III) facilitates SET, ^3^EnT, or both when combined with Ni(II)–bpy aryl halide is at the core of the Photosensitization for Homolysis mechanisms shown in Figures 5–6. It is evident from Stern–Volmer analysis that Ni(II)–bpy aryl halide complexes do quench ^3^Ir(III) excited states (*vide supra*), but the mechanism of quenching is still undetermined.

Reports by MacMillan, Scholes, and co-workers on Ni(II)–bpy aryl acetate complexes favor ^3^EnT over reductive SET,^97^ but it is possible that halide to acetate ligand exchange sufficiently alters the electronic structure of the compound to favor one mechanism vs another. Mechanism switches have been reported for Ni(II)–polypyridyl complexes upon halide to acetate ligand substitution.^83^ Moreover, reduced reactivity was observed by Molander and co-workers when employing strongly oxidizing photocatalysts that were unproductive for ^3^EnT,^98^ a result that contrasts the good product yields observed by MacMillan and co-workers when using an external chemical oxidant in place of the photosensitizer.^97^ Beginning with Ni(0) and aryl halide, Rueping and co-workers^102^ proposed a similar mechanism to that proposed by Molander and co-workers (Figure 6). In this case, strongly oxidizing photocatalysts also did not provide good product yields, but neither did direct excitation of the independently synthesized Ni(II)–bpy alkyl bromide complex. Notably, no direct evidence for Ni–X homolysis was provided in this work.

In related reports, Ni(II)–bpy acyl chlorides were examined by Shibasaki and co-workers^103^ in 2017; they found photosensitizers with high triplet energies and low oxidizing power alone gave good product yields (favoring ^3^EnT Photosensitization for Homolysis). However, Paixão, König and co-workers^104^ surmised that beginning with a high-spin Ni(II)–bpy dihalide precursor gave entry via SET into the Photosensitization for Homolysis mechanism (Figure 5) and not the ^3^EnT pathway (Figure 6). Notably, alternative routes have been demonstrated for the combination of Ni(II)–bpy dihalide and photocatalyst. *Therefore, the electronic structure of the receiving low-spin Ni(II)–bpy complex is susceptible to changes by both ligands, the aryl and the halide, and it is possible that high-spin Ni(II)–bpy dihalide precursors can enter into a variety of pathways upon photosensitization.* One should take care when extending mechanistic analysis of one Ni complex to even seemingly similar ones. Careful experimental electronic structure-centered analysis on the mechanism of excited-state quenching between Ni(II)–bpy aryl halide and *Ir(III) is still needed.

##### 3.1.6.3 Halide Radicals Are Photogenerated

Mechanisms outlined in Figure 5–6 both rest on a critical Ni–X bond homolysis induced via the photosensitizer, either through SET or ^3^EnT. Kinetic isotope effect measurements supported the generation of radicals, with halide radicals being favored over aryl radicals by Doyle, Shields, and co-workers.^57,105^ Evidence of aryl radical generation upon direct excitation of Ni(II)–bpy aryl halide complexes has also been provided on numerous accounts (pathway discussed in Section 3.2.1). Evans–Polanyi analysis conducted on the Photosensitization for Homolysis pathway by Doyle and co-workers in 2018 determined an α-value of 0.44,^106^ near that for the proposed Cl^•^ (α_Cl_ = 0.45), but also near CH_3_^•^ (α_CH3_ = 0.45), and H^•^ (α_H_ = 0.43);^107^ the α_C6H5_ value is unknown. Discrete experiments using Ni(II)–bpy aryl halide in conjunction with chemical oxidant and light source found that 1) the dehalogenated arene, Ar–H, is produced in 40% yield, 2) the direct reductive elimination product, Ar–X, is produced in 12% yield, 3) the solvent-aryl cross-coupled product is produced in 7% yield, and 4) the bis-aryl is produced 2% yield.^36^ These values, in particular the large amounts of Ar–H, are suggestive of aryl radical generation, not halide radicals. These disparate conclusions call for more detailed work to evaluate these proposed mechanisms.

##### 3.1.6.4 Reductive Elimination Proceeds from a Ni–bpy Aryl Alkyl Species

In Figure 5, cross-coupled product is the result of reductive elimination from an oxidized [Ni(III)–bpy aryl alkyl]^+^ complex. Indeed, reductive elimination from more highly oxidized Ni complexes is well established.^91,92^ However, the direct Ni(II)–Ni(0) reductive elimination in Figure 6 is less common. Stable Ni(II)–bpy aryl alkyl complexes have been synthesized and isolated by Park and co-workers; reductive elimination does not proceed under irradiation or at elevated temperatures (75 °C).^108,109^ Thus, it remains unclear whether Ni(II)–Ni(0) C(sp^2^)–C(sp^3^) reductive elimination is thermodynamically feasible near room temperature.

We note that, shortly before finalizing this Review, a mechanistic study by Doyle and co-workers^110^ was deposited to the ChemRxiv preprint server. In this detailed work, the authors revisit the above-mentioned 2016 proposals, finding that 1) ^3^EnT from *Ir(III) to Ni(II)–bpy aryl halide promotes reductive elimination of the aryl halide. 2) Direct light absorption by a Ni(II)–bpy aryl halide complex affords an aryl radical and Ni(I)–bpy halide; photohalogen elimination from the Ni(II) complex is not favored. 3) In addition to aryl radical generation, excitation of the Ni(II) complex likely also facilitates excited-state reductive elimination of the aryl halide to afford Ni(0)–bpy. 4) *In situ* oxidation to [Ni(III)–bpy aryl halide]^+^ immediately releases the aryl halide at room temperature. However, at cryogenic temperatures, the Ni(III) species persists long enough to absorb an additional photon, again ejecting an aryl radical and not the halogen, in agreement with previous computational work.^14^ 5) C(sp^2^)–C(sp^3^) reductive elimination from a Ni(II)–bpy aryl alkyl species is faced with a substantial room temperature barrier of ∼25 kcal mol^–1^. However, absorption of high energy light (390–470 nm) by this complex promotes the formation of aryl–alkyl product. This mechanistic work illustrates the importance of thorough experimental consideration of each step in proposed mechanistic cycles, such as those presented in the Key Points highlighted above.

#### 3.1.7 Triplet Energy Transfer (^3^EnT)

**Figure 7:**
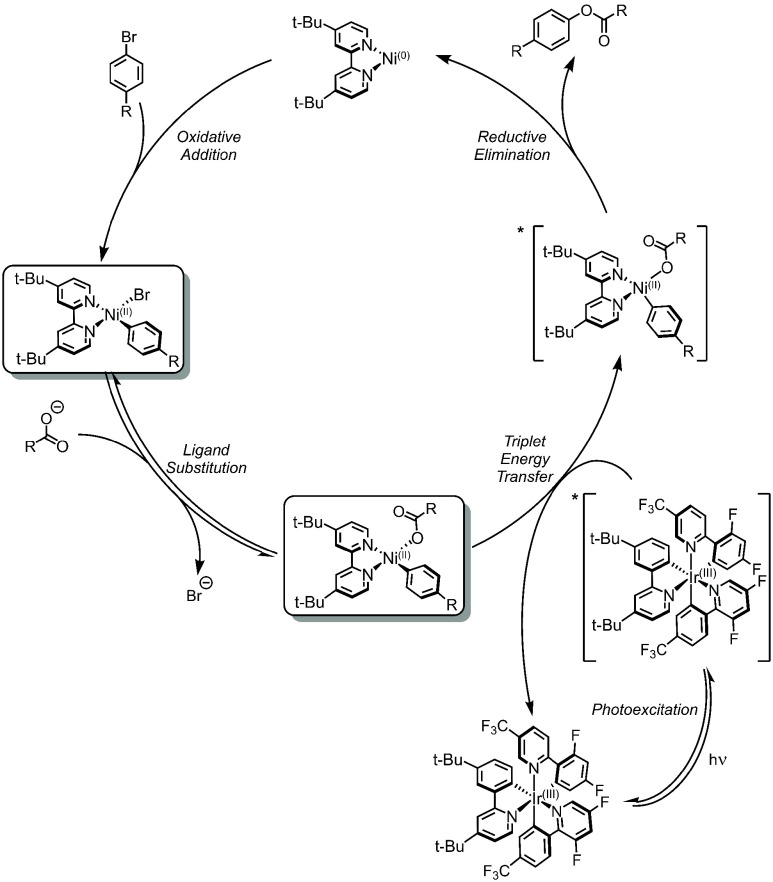
Proposed ^3^EnT Mechanism. C(sp^2^)–O coupling (carboxylic acids) coupling is shown as a representative example.

In 2017, McCusker, MacMillan and co-workers demonstrated C(sp^2^)–O cross-coupling of aryls and carboxylic acids;^96^ product yields correlated with the ^3^EnT ability of the photocatalyst, not its oxidizing potential, a result that argued against the Oxidative SET mechanism outlined in Figure 4. Rather, a ^3^EnT mechanism was proposed (Figure 7).

The ^3^EnT mechanism also features oxidative addition of the aryl halide to Ni(0)–bpy to form a Ni(II)–bpy aryl halide species (left box, Figure 7). Base assisted ligand substitution generates a Ni(II)–bpy aryl acetate species (bottom box, Figure 7). The Ir(III) complex acts as the primary light-absorbing species to form *Ir(III). This excited state is quenched by the Ni(II)–bpy aryl acetate complex through Dexter EnT (*k*_q_ = 10^9^ M^–1^ s^–1^ by Stern–Volmer analysis)^95,98^ affording ground-state Ir(III) and an excited-state Ni(II) complex, *[Ni(II)–bpy aryl acetate]. Reductive elimination was proposed to occur from a Ni(II)-based ligand field excited state, resulting in C(sp^2^)–O cross-coupled product and Ni(0)–bpy.

#### 3.1.8 Key Components of the ^3^EnT Mechanism

##### 3.1.8.1 Ir(III) Is the Primary Light-Harvesting Species, but Potentially Not the Only One

While it is clear from Stern–Volmer analysis that the Ni(II)–bpy aryl acetate quenches *Ir(III), excitation without the photosensitizer also results in cross-coupled product (albeit with slower kinetics under the given conditions).^96^ Furthermore, the precursor Ni(II)–bpy aryl halide species also absorbs light;^37^ direct excitation of Ni(II)–bpy aryl halide in the presence of cross-coupling partners is productive for C–O bond formation (discussed in Section 3.2.2),^36,79^ marking at least three distinct light-harvesting species present in the reaction mixture (Figure 2). It is unclear if one or more mechanisms are operative. However, given the photosensitizer’s high absorption cross-section and quantum yield for ^3^Ir(III) formation,^101^ its excitation may be dominant.

##### 3.1.8.2 ^3^EnT, Not SET, Is the Dominant Mechanism Promoting Photosensitized Cross-Coupling Reactions

The proposed divergence away from SET to favor ^3^EnT for C(sp^2^)–O coupling seems contingent upon the presence of the Ni(II)–bpy aryl acetate species.^111^ Previous reports favored SET to the Ni(II)–bpy aryl halide precursor (*vide supra*, Section 3.1.3). However, several observations support ^3^EnT from *Ir(III) to the Ni(II)–bpy aryl acetate species: 1) the lack of reactivity with a chemical reductant, 2) a ^3^EnT threshold for product yield of ∼40 kcal mol^–1^, 3) product generation upon direct excitation of the Ni complex alone, and 4) an inverse correlation between product and oxidizing power of the photocatalyst.^96^ Furthermore, computational analysis by Chen and co-workers^13^ and follow-up ns transient absorption studies on C(sp^2^)–O coupling reactivity by MacMillan, Scholes, and co-workers^97^ demonstrated additional support for the ^3^EnT mechanism, although in the latter, chemical oxidant was still effective for product yield. Recent work by Oderinde, Hudson, and co-workers finds that a series of organic ^3^EnT photosensitizers are also competent photocatalysts for C(sp^2^)–O esterification reactions when used in combination with Ni(II)–bpy aryl acetate species.^112^*It may be the case that both the aryl halide and aryl acetate complexes undergo*^3^EnT with *Ir(III), but only in the case of the aryl acetate is it irreversible and productive for catalysis.

The aryl halide coupling partner has also been implicated as redox noninnocent. Pieber, Seeberger, and co-workers^113^ conducted a kinetic analysis of the aryl–acetate C(sp^2^)–O coupling presented by McCusker, MacMillan and co-workers in 2017, but they began with Ar–I instead of the Ar–Br substrates. Evidence supported rapid SET from *Ir(III) to Ar–I; this off-cycle electron transfer, which resulted in a dehalogenated Ar–H product, was said to be involved in turnover-limiting oxidative addition of the substrate to Ni(0). However, the Ni precursor used was Ni(II)–bpy dihalide, not Ni(0), making the presence of Ni(0) somewhat speculative (see Point 3 below). Nonetheless, the authors cited previous work to propose that ^3^EnT between *Ir(III) and Ni(II)–bpy aryl acetate was the active mechanism for C–O coupled product formation.^113^

##### 3.1.8.3 The Proposed Cycle Rests on the Ni(0)–bpy/Ni(II)–bpy Aryl Halide Combination as the Starting Source of Nickel

As in the Oxidative SET Mechanism, the reaction was initiated with a Ni(0) source to give the Ni(II)–bpy aryl halide precatalyst. However, optimized reaction conditions utilized high-spin Ni(II)–bpy dihalide as the precursor nickel source.^96^ The same complication is then introduced in this ^3^EnT proposal. It is unclear if the experimental mechanistic analysis conducted between the Ir photosensitizer and the Ni(II)–bpy aryl acetate complex holds true for the Ir and Ni(II)–bpy dihalide combination. Important and complementary analysis was conducted by Li, Huang, Zhang and co-workers^114^ in 2018 on the aryl acetate coupling reaction but using organic photosensitizers in place of the Ir(III) and beginning with Ni(II)–bpy dihalide. Under these conditions, strongly oxidizing photocatalysts gave an undesired dehalogenation of the aryl halide and no cross-coupled product, consistent with the 2017 study.^96^ Transitioning to ^3^EnT-active photosensitizers with lowered oxidation potentials gave some product yield, but still saw ∼20% dehalogenation product. Thus, it was reasoned that when using Ni(II)–bpy dihalide as the precursor species, SET is favored over ^3^EnT and proceeds prior to energy transfer.^114^ Only careful and deliberate suppression of oxidation allows for ^3^EnT to become the major pathway. Under standard conditions (i.e., with Ir(III)), SET is therefore likely the sole or dominant mechanism, but it is unclear from these studies if the oxidation occurs with Ni, with the substrate coupling partners, or with the exogenous amine base. This electron transfer event is explicitly considered in the SET for Active Ni(I) Mechanism (Section 3.1.9), a proposal that appears to be the principal pathway when combining Ir(III) and Ni(II)–bpy dihalide, thereby marking a critical mechanistic switch when using high- vs low-spin Ni(II) precursors.

It is clear from these studies that the highly potent and versatile *Ir(III) may engage in multiple pathways at once, including ^3^EnT and SET. Diversion from one route to another depends on the Ni catalyst precursor, substrate coupling partners, exogenous base, and even photon intensity.^58,115^

#### 3.1.9 SET for Active Ni(I)

**Figure 8:**
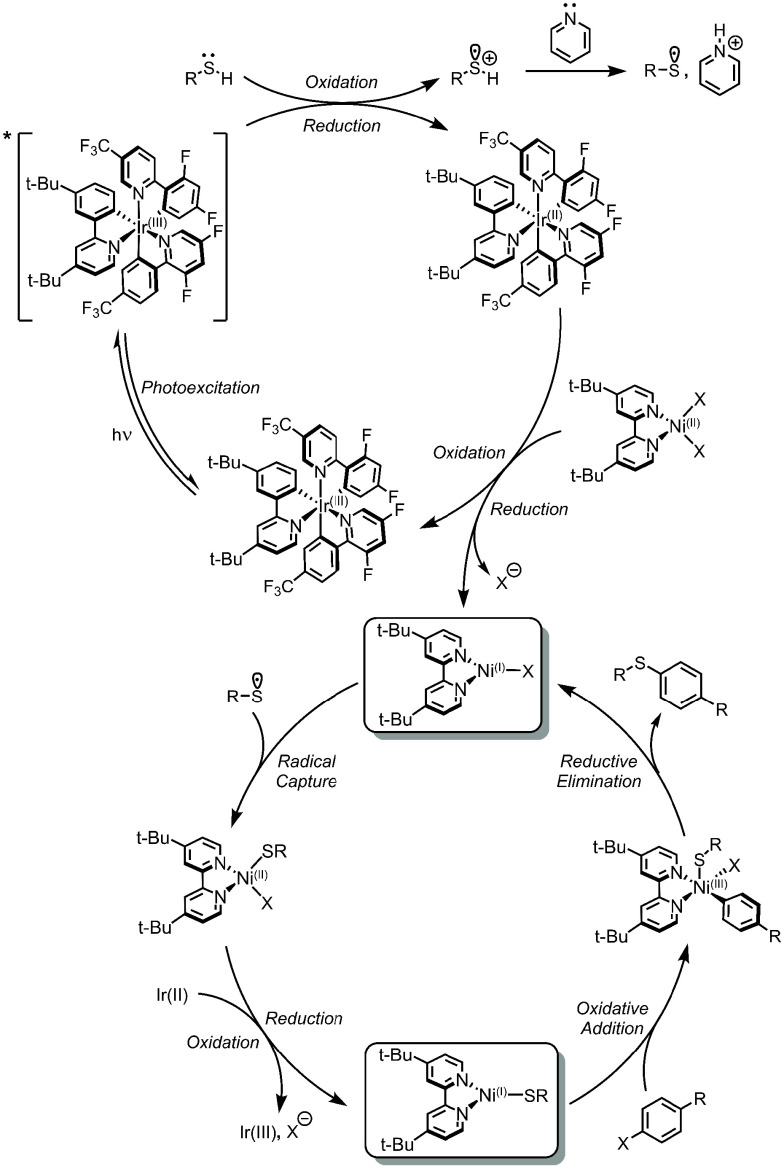
Proposed SET for Active Ni(I) Mechanism for C(sp^2^)–S coupling.^69^

Dual Ir/Ni cross-coupling reactions beginning with Ni(II)–bpy dihalide precursors have seen wide use, largely due to their incredible substrate scope potential. In 2016, Oderinde, Johannes, and co-workers uncovered C(sp^2^)–S coupling reactivity by combining an aryl halide and thiol with Ni(II)–bpy dichloride and Ir(III).^69^ Interestingly, it was found through Stern–Volmer analysis and radical traps that the thiol appreciably quenches *Ir(III) (*k* = 10^5^ M^–1^ s^–1^) to generate thiyl radicals and Ir(II). Ir(II) reduces the high-spin Ni(II)–bpy dihalide complex, affording Ni(I)–bpy halide and Ir(III) (Figure 8).

The Ni(I)–bpy halide is at the core of the catalytic cycle turnover. First, it is intercepted by the thiyl radical to make a Ni(II)–bpy halide sulfide complex, which is reduced by a second equivalent of Ir(II) to Ni(I)–bpy sulfide. This Ni(I) undergoes oxidative addition with aryl halide to form a Ni(III)–bpy aryl halide sulfide species. This complex undergoes rapid reductive elimination to return Ni(I)–bpy halide and the C(sp^2^)–S coupled product (Figure 8).^116^ The generalizability of the reaction became evident by the extension to C(sp^2^)–N coupling of aryls and amines.^117^

Inspired by the 2018 work of Miyake and co-workers,^118^ which demonstrated the formation of arylamines upon direct excitation of a nickel-amine complex formed *in situ* form Ni(II)Br_2_, Neurock, Minteer, Baran, and co-workers^55^ eliminated the photocatalyst, demonstrating that the Ni(II)–Ni(I) initiation step could be achieved through applied electrochemical potential. As discussed in the Ni Electronic Structure Primer (Section 2), the high-spin Ni(II)–bpy dihalide has a less negative (more accessible) reduction potential than a Ni(II)–bpy aryl halide species, an additional species formed in the catalytic cycle. By controlling the applied potential, the researchers ruled out Oxidative SET; electrochemical oxidation of Ni(II)–bpy aryl halide did not occur within the solvent window. Ni(I)–bpy halide intermediates were observed spectroelectrochemically and were demonstrated to be active toward oxidative addition, thereby facilitating reaction turnover.^55^

**Figure 9:**
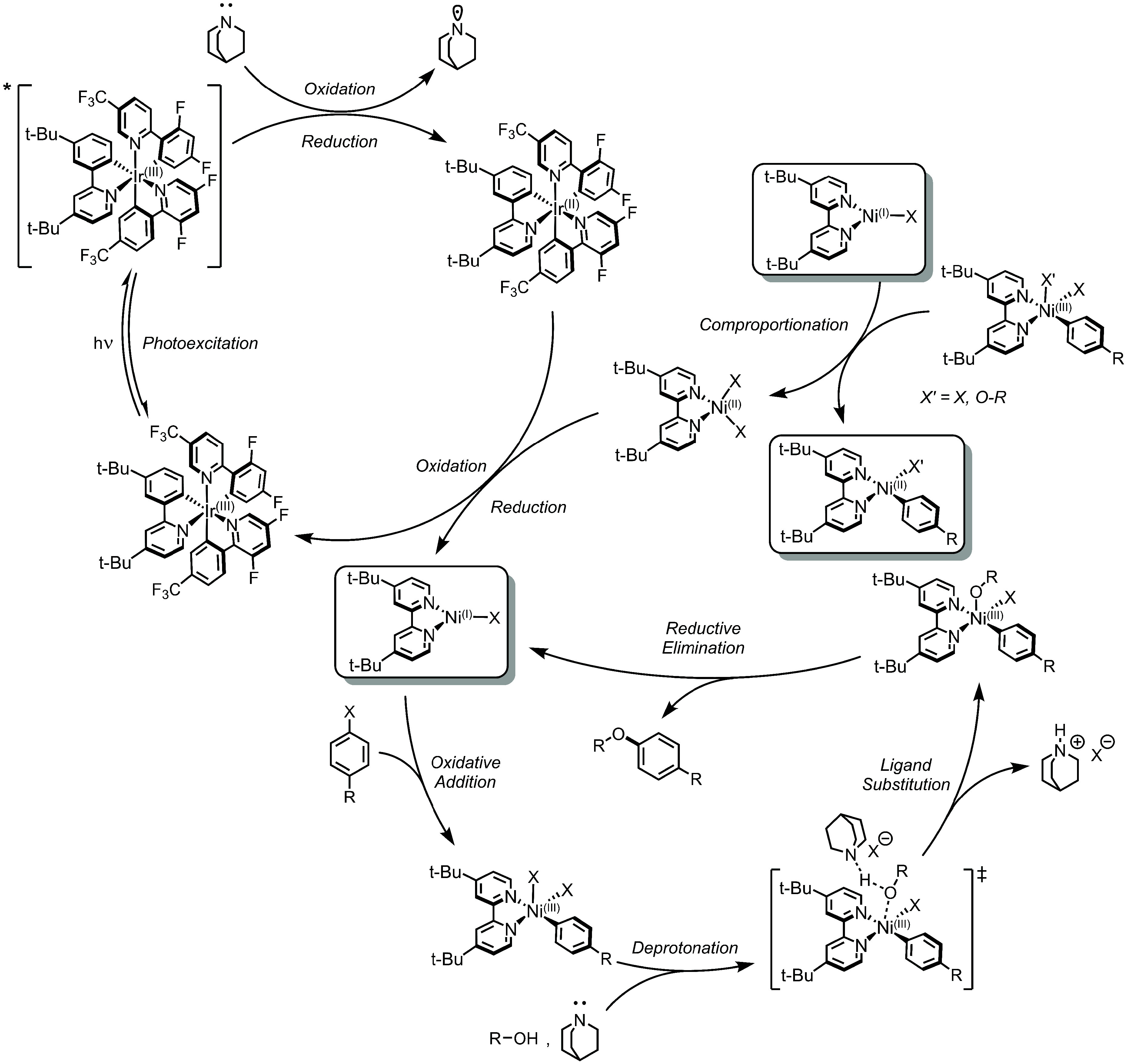
Proposed SET for Active Ni(I) Mechanism. C(sp^2^)–O coupling (alcohols) is shown as a representative example.

While examining the C(sp^2^)–O coupling of aryl halide and alcohol coupling partners pioneered by MacMillan and co-workers^28^ in 2015 (see Oxidative SET, Figure 4, above), Nocera and co-workers^95^ instead found support for the SET for Active Ni(I) mechanism (Figure 9). Again, Ir(III) is the sole light-harvesting species, being promoted to *Ir(III). Like in the case of aryl thiolate formation, the exogenous base used for the ligand substitution step (i.e., quinuclidine), was found to quench *Ir(III) with ease, generating Ir(II) and amine cation radicals. Ir(II) reduces the Ni(II)–bpy dihalide to Ni(I)–bpy halide and Ir(III). This Ni(I) species undergoes oxidative addition to form a Ni(III)–bpy aryl dihalide species, followed by ligand substitution of the halide by the alcohol/alkoxide and subsequent reductive elimination of the C(sp^2^)–O product. However, it was also proposed that the Ni(I)–bpy halide species can be diverted via comproportionation with the Ni(III)–bpy aryl dihalide species to regenerate the *S* = 1 Ni(II)–bpy dihalide complex and the *S* = 0 Ni(II)–bpy aryl halide species. The former acts to regenerate the cycle, while the latter presents an off-pathway sink for diminished catalysis.

Indeed, the Ni(II)–bpy aryl halide itself can aggregate with the Ni(I)–bpy halide, as observed by Nocera and co-workers^95^ (using X-ray crystallography and EPR), as well as Hadt and co-workers^56^ (using temperature-dependent spectroscopic methods).

Following these three studies, researchers began to revisit previous mechanistic proposals. In 2020, MacMillan and co-workers^67^ conducted a detailed study on the mechanism proposed in 2016 for C(sp^2^)–N coupling^90^ (see Oxidative SET, Figure 4) and found the SET for Active Ni(I) Mechanism was better supported by their data, despite using the amine base DABCO (1,4-diazabicyclo[2.2. g2]octane) instead of quinuclidine. DABCO quenches *Ir(III) with a quantum yield near unity (Φ > 0.99) and near diffusion-controlled kinetics (*k* = 10^9^ M^–1^ s^–1^), affording amine^•+^ and Ir(II). This initial quenching step was confirmed using spectroelectrochemistry and transient absorption spectroscopy and was not found to be sensitive to the addition of either aryl halide or high-spin Ni(II)–bpy dihalide. When varying the photosensitizer, the data further indicated that SET from Ir(II) to Ni(II) was involved in the rate-determining step of the overall catalytic cycle.^67^

#### 3.1.10 Key Components of the SET for Active Ni(I) Mechanism

##### 3.1.10.1 Ir(III) Is the Sole Light-Harvesting Species, and Its Excited State Is Quenched by Exogenous Organic Base (e.g., Amine or Thiol in Solution) to Generate Ir(II)

*Ir(III) is quenched by the amine or thiol in solution (Figures 8–9); this fact has been verified by numerous sources via Stern–Volmer analysis.^67,95,118−120^ In the case of C(sp^2^)–S coupling, 12 discrete rate constants have been elucidated, altogether pointing to a self-sustained Ni(I)/(III) cycle with product Φ > 1.^119^ Nocera and co-workers also demonstrated that Ni(II)–bpy dihalide is an effective quencher of *Ir(III), but with a quenching rate constant ∼ six times smaller than that of quinuclidine.^95^ Interestingly, the Ni(II)–bpy aryl alkoxide complex generated by comproportionation of Ni(I) and Ni(III) also quenches *Ir(III), but with a rate constant twice as large as that of quinuclidine.^95^*It is therefore possible that the cross-coupling reaction begins with the SET for Active Ni(I) Mechanism, but once sufficient concentration of the Ni(II)–bpy aryl alkoxide species is generated, the mechanism diverts to one in which the* S *= 0 Ni(II) species is the dominant quencher (e.g., the*^3^*EnT mechanism discussed above).* Computational evidence by Liu, Tlili, and by Zhu and Guan and their co-workers lends preliminary support to this hypothesis.^121,122^

A switch in mechanism, or multiple, simultaneous kinetically competing mechanisms occurring in dual Ir/Ni(II)–bpy dihalide catalysis is likely. MacMillan found that C(sp^2^)–N coupling for arylamines proceeded rapidly with DABCO present, but it was not switched off with DABCO absent; the reaction rate decreased by ∼ nine times as a function of decreasing DABCO concentration, but 14% product yield was still obtained without DABCO.^67^ Thus, the more kinetically active mechanism involves the quenching of the amine, but *Ir(III) is also quenched by other species present in solution (including high-spin Ni(II)–bpy dihalide). Discrete reactivity pathways with *Ir(III) and Ni(II)–bpy dihalide in the absence of other quenchers were also examined, finding that no Ir(II) spectral features were observed without the organic quencher and, thus, invoking another cycle that does not involve the reduced Ir(II) species as an intermediate.^67^ This analysis was computationally extended by Su, Guan, and co-workers,^123^ then experimentally by Escobar, Thordarson, Johannes, Miyake, and co-workers.^124^ It was proposed through ns transient absorption spectroscopy and oxidative spectroelectrochemistry that *Ir(III) is oxidatively quenched by high-spin Ni(II)–bpy dihalide to give Ni(I)–bpy halide and Ir(IV);^124^ computations by Su in 2018 also favor this pathway.^125^ The Ir(IV) is reduced downstream to return the starting Ir(III) by SET from a redox noninnocent aryl halide substrate.^124^

##### 3.1.10.2 Ni(II)–bpy Dihalide Is the Precursor Source of Ni and Is Reduced by Ir(II) to Ni(I)–bpy Halide

The reduction of Ni(II) to Ni(I) by Ir(II) has been demonstrated multiple times (*vide supra*). Additionally, the importance of Ni(I) for product yields was established through a photosensitizer screen by MacMillan and co-workers.^67^ Analysis of a library of Ir(III) photocatalysts demonstrated that product yields and reaction rates trend with Ir(II) reduction potential. However, the Ir(II) reduction potential also trends well with ^3^EnT capability. The first nonfunctioning photosensitizer has an emission energy of ∼45 kcal mol^–1^ and a reduction potential of −0.77 V vs SCE. This energy transfer threshold is similar to that used to support ^3^EnT,^96^ which makes these trends alone a poor distinction between mechanisms. However, the first functioning photosensitizer (albeit with low product yields of ∼3%) makes a notable exception to this trend. It has an emission energy of ∼62 kcal mol^–1^ and reduction potential of −1.23 V vs SCE. The best performing Ir photocatalyst (100% yield) has a ^3^EnT potential of 61 kcal mol^–1^ but reduction potential of −1.91 V vs SCE, confirming that reduction potential, not ^3^EnT energy, is a good predictor of productive catalysis.^67^ In accord, Rovis and co-workers utilized a red light absorbing Os(II) photocatalyst with a highly reducing potential to successfully achieve C(sp^2^)–N coupling.^126^ Therefore, Ni(I) formation is on-pathway and required for product formation. This result was further supported by Nocera and co-workers, who found an induction period when organic quenchers were not present to transform *Ir(III) to Ir(II), the preferred species for Ni(II) to Ni(I) reduction,^119^ and by Liu, Tlili, and co-workers who leveraged active Ni(I) for C(sp^2^)–N coupling with CO_2_ as electrophile.^121^

##### 3.1.10.3 Ni(I)–bpy Halide Supports a Dark Ni(I)/(III) Cycle

The reactivity of Ni(I)–bpy halide toward oxidative addition and subsequent reductive elimination is well established.^70,127^ Recent mechanistic studies have identified rate constants for the reaction between Ni(I)–bpy halide and aryl iodides, bromides, and chlorides.^56,60,82,105^ The immediate product of this reaction has been confirmed as Ni(III) by comparison to model complexes.^82,128^ Importantly, Ni(I)–bpy halide species undergo dimerization or oligomerization to binuclear or polynuclear Ni species, respectively, representing significant off-cycle deactivation pathways.^56,82,95,129^ Because of the exponential rate law dependence on the Ni(I) concentration in these reactions, maintaining lower Ni(I) concentration leads to improved cross-coupling yields. Indeed, by modulating the flux of the incident light to minimize the rate of *Ir(III) formation (and therefore the downstream concentration of Ni(I) at a given time), the quantum yield for product formation could be increased ∼15 times (from Φ = 1.6 to Φ = 25).^95^ The observation of a quantum yield greater than one at even high flux levels supported a dark Ni(I)/(III) cycle. A similar observation was made for aryl thiolate formation.^119^

The prevalence of the SET for Active Ni(I) Mechanism cannot be overstated. König reports a general reaction beginning with high-spin Ni(II)–bpy dihalide that is competent for C(sp^2^)–X (X = C(sp, sp^2^, sp^3^), S, Se, N, O, P, B, Si, Cl) coupling.^130^*Mechanistic work is needed on a case-by-case basis, but the authors propose that this dramatic substrate scope is largely, if not fully, dominated by a Ni(I)/(III) self-sustaining cycle, even when alternative mechanisms are possible.*

### 3.2 Direct Excitation

This section, divided into two parts, describes research invoking direct photon absorption by specific Ni-based species involved in catalytic cycles. The first part considers cases where direct photoexcitation generates Ni-based intermediates for dark reactions that mediate cross-coupling. The second considers cases in which the cross-coupling events occur directly from transient excited states.

#### 3.2.1 Direct Excitation for Dark Cycle Initiation

##### 3.2.1.1 Photoinduced Ni–X Bond Homolysis

**Figure 10:**
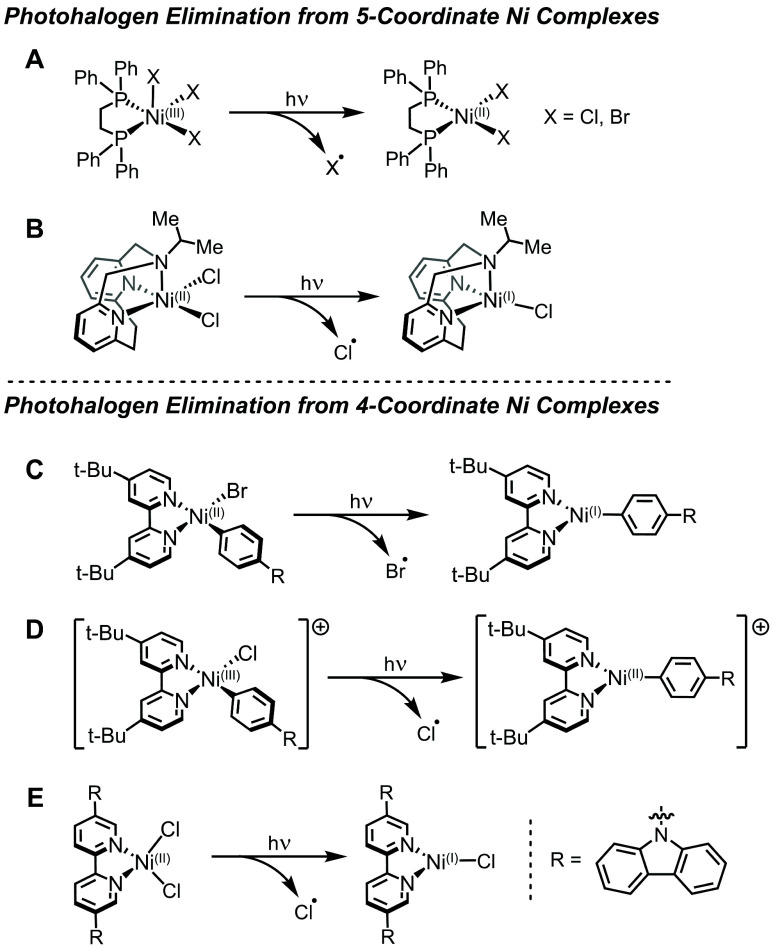
Proposed direct excitation for photohalogen elimination from five-coordinate Ni complexes studied by (**A**) Nocera^99,100^ and (**B**) Mirica,^128^ and from four-coordinate Ni complexes studied by (**C**) Molander,^98^ (**D**) Doyle, Shields,^57^ and (**E**) van der Veen, Thomas, and Pieber.^131^

Photoinduced Ni–X bond homolysis has been proposed as an initiation step that generates reactive species involved in key dark reactions. Often drawing comparisons to photohalogen elimination from five-coordinate Ni complexes, researchers have proposed pathways for metal–halide bond homolysis from four-coordinate Ni (Figure 10). These include the generation of triplet excited-states of Ni(II)–bpy aryl halide species, photohalogen elimination from a Ni(III)–bpy aryl halide intermediate, the generation of intraligand charge transfer excited states that relax to dissociative metal-based excited states, and the direct generation of photoactive triplet metal-centered excited states of Ni(II) complexes with triplet ground states.

In 2016, Molander and co-workers reported C(sp^3^)–H arylation using a Ni(II)–bpy aryl bromide catalyst^98^ (Section 3.1.5, Figure 4; Figure 10C). Arylated product was observed when the reaction was carried out with an Ir(III) photosensitizer, as discussed above. However, it was noted that arylated product could be detected when the reaction mixture was irradiated at specific wavelengths without the Ir(III) photosensitizer. Visible light excitation (∼400–600 nm) did not lead to product, while UV–B irradiation (290–315 nm) did. Based on these results and additional control photosensitization experiments, a catalytic mechanism was proposed that included a triplet excited state responsible for Ni reactivity. Mechanistic scenarios were presented for the generation of reactive Ni intermediates, all of which involved Ni(II)–Br bond homolysis. In the case of direct UV–B excitation, a high-energy singlet excited state was proposed to relax via nonradiative intersystem crossing to a triplet excited state from which the Ni(II)–Br homolysis could occur.^98^ As discussed above, the resultant bromine radicals were suggested to activate THF through hydrogen atom abstraction, and coupling to the aryl ligand could occur via reductive elimination from Ni(II) in an overall Ni(0)/Ni(II) cycle (see Figure 4).

Also in 2016, Doyle and co-workers reported a C(sp^3^)–H cross-coupling platform with Ni(II)–bpy aryl chloride and an Ir(III) photocatalyst^57^ (Section 3.1.5, Figure 4; Figure 10D). As discussed above, the Ir(III) photocatalyst is proposed to carry out reductive SET to generate Ni(0) species for oxidative addition with the aryl chloride, as well as oxidative SET to oxidize the Ni(II)–bpy aryl chloride. The resulting cationic [Ni(III)–bpy aryl chloride]^+^ intermediate was proposed to undergo direct photon absorption to drive excited-state Ni(III)–Cl bond homolysis via an LMCT state in a manner analogous to an isolable pentacoordinate Ni(III) species^99,100^ (Figure 10A).

Ni(II)–X bond homolysis was further proposed in a Ni(II)–bpy dihalide *S* = 1 system that featured a dicarbazolyl functionalized bpy ligand (Figure 10E).^131^ The extended ligand alters the bpy orbital energies levels such that intraligand charge transfer (ILCT) states are present in the visible region. Combining transient absorption with computational analysis, the mechanism of Ni(II)–Cl bond homolysis was proposed to involve an initial excitation into a ^3^ILCT state, followed by relaxation into an optically dark square-planar metal-centered state. This state was proposed to feature antibonding character along the Ni–halide bond, thereby facilitating Ni(II)–X bond homolysis and formation of catalytically relevant Ni(I) species.

##### 3.2.1.2 Photoinduced Ni–C Bond Homolysis

**Figure 11:**
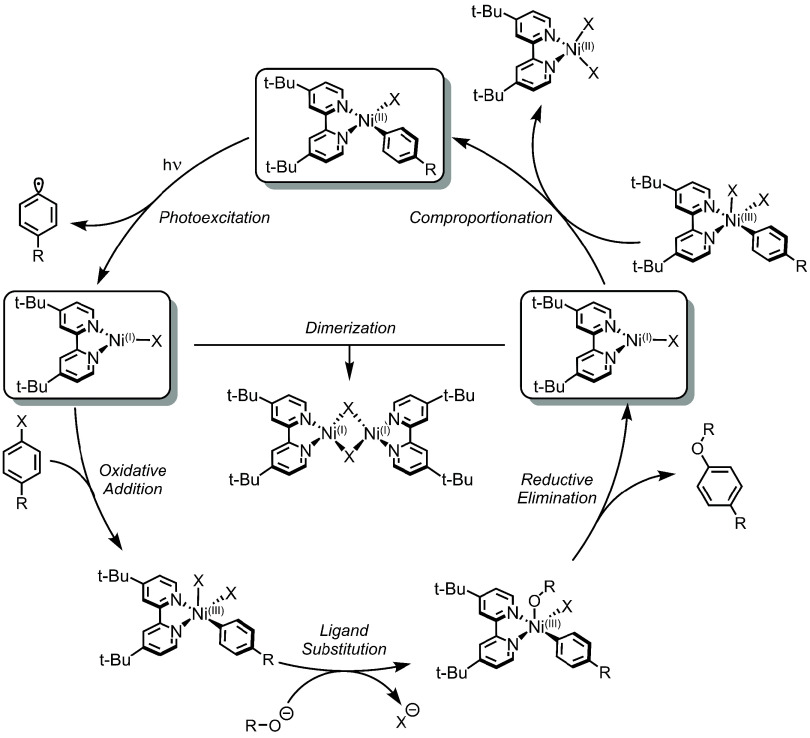
Proposed direct excitation for Ni(II)–C(aryl) bond homolysis and C(sp^2^)–O product formation mechanism (as a representative example). Following light-initiation, Ni(I)–bpy halide participates in “dark” substrate turnover but can be deactivated via off-cycle dimerization.

In addition to excited-state Ni–X bond homolysis, recent studies have invoked analogous Ni–C bond homolysis.^36−38^ For Ni(II)–bpy complexes, after photoexcitation and carbon radical formation, the resultant Ni(I)–bpy halides mediate dark chemistry leading to the cross-coupled products (including C(sp^2^)–C(sp^3^), O, N, S coupling)^36,79,109,132,133^ via the proposed mechanism outlined in Figure 11.

In 2018, Doyle and co-workers utilized transient absorption spectroscopy to study the excited-state dynamics of isolable Ni(II)–bpy aryl halide compounds.^36^ It was proposed that the excitation of the complex resulted in ^3^MLCT excited states that could undergo bimolecular electron transfer with a ground-state Ni(II)-bpy aryl halide species. The downstream result of this photochemical process was aryl ligand loss and the generation of a three-coordinate Ni(I)–bpy halide that would engage in Ni(I)/Ni(III) oxidative addition/reductive elimination cycles for C(sp^2^)–O cross-coupled product formation. However, later studies conducted by MacMillan, Scholes, and co-workers^97^ indicated that this bimolecular photoinduced disproportionation pathway is not operative, as Stern–Volmer studies did not find appreciable Ni(II) excited-state quenching by ground state Ni(II)–bpy aryl halide.

In a subsequent 2020 study, Doyle and co-workers expanded on their earlier transient absorption analysis and proposed an alternative excited-state relaxation pathway that could lead to Ni(II)–C(aryl) bond homolysis via a triplet ligand field (^3^d-d) excited state of Ni(II)–bpy aryl halides.^37^ Transient spectroscopic measurements carried out with either a 530–590 nm (for transient absorption) or 610 nm (time-resolved IR) laser pump demonstrated that initial excitation dominantly populates a ^1^MLCT excited-state manifold, which can relax through additional MLCT states to ultimately form the ^3^d-d state. By correlating DFT calculations to transient absorption and 2D exchange NMR experiments, it was proposed that the ^3^d-d state features a pseudo-*T*_d_ geometry (see Figure 1B) that can be accessed photochemically or thermally at room temperature. This pseudo-*T*_d_ geometry featured electron population of a σ* orbital, reducing the Ni-aryl bond order to one-half, thereby activating it for thermally driven homolysis. In support of that, DFT predicted a significantly weaker Ni(II)–Cl bond in the ^3^d-d excited state (∼24 kcal mol^–1^) vs the ground state (∼35 kcal mol^–1^). Both ^1^H NMR and EPR spectroscopy confirmed the generation of aryl radicals; no chlorine radicals were trapped, arguing against photoinduced Ni(II)–Cl homolysis.^37^

Also in 2020, Hadt and co-workers explored mechanistic aspects of excited-state Ni(II)–C(aryl) bond homolysis from Ni(II)–bpy aryl halides using quantum chemical calculations of both ground- and excited-state PESs.^12^ Multireference/multiconfigurational calculations suggested intractable energies for thermal bond dissociation from the lowest-energy ^3^d-d state, with calculated bond strengths differing significantly from those predicted by DFT. This study also suggested an alternative mechanism of excited-state Ni(II)–C(aryl) bond homolysis that featured 1) initial ^1^MLCT formation and 2) intersystem crossing and aryl-to-Ni LMCT to form repulsive triplet excited-state PESs.^12^ These MLCT/LMCT surfaces featured a Ni(II)–C(aryl) σ → σ* electron excitation, which reduces the bond order to zero, hence the repulsive excited-state PESs. Notably, such description of dissociative excited-state bond homolysis conceptually resembles the isolable pentacoordinate Ni(III) photochemistry,^99^ as well as the mixed MLCT/σπ* (σ bond to ligand charge transfer) photoinduced radical formation in Re(I) and Ru(II) complexes.^134−138^

In 2021, Park and co-workers proposed an analogous mechanism, utilizing Ni(II) complexes with cyclic ligands inherently predisposed to facile photochemical reductive elimination.^109^ Regardless of the bidentate backbone ligand (diimine, diamine, or diphosphine), all studied complexes exhibited photoactivity under irradiation.

The ^3^LMCT excited-state PES was suggested to initiate carbon radical generation. This could occur through pathways from a preferred ^1^MLCT state in aromatic diimine complexes or from a ^1^d-d state in aliphatic diamine or diphosphine complexes lacking low-lying unoccupied ligand-based orbitals. Additionally, both ^1^d-d and ^1^MLCT excited states may operate simultaneously, with their ratio depending on the ground-state complexes’ electronic structure and molar absorptivities. Importantly, charge transfer excitations exhibit orders of magnitude higher molar absorptivity than ligand field transitions, but vibronic coupling with a weakly absorbing, dissociative triplet state can potentially mediate intersystem crossing and Ni–C bond homolysis.

Following their earlier work, Hadt and co-workers provided further exploration of the excited-state bond homolysis mechanism.^38^ In 2022, experimental analysis of a library of Ni(II)–bpy aryl halides found that 1) Ni(II)–C(aryl) bond homolysis was dependent on the bpy (MLCT acceptor) and aryl (LMCT donor) ligand substituents. A linear relationship was found for bpy/aryl Hammett parameters^139^ and the rate constants and quantum yields for photochemical aryl radical generation; switching the halide from Cl to Br to I increased the rate of Ni–C bond homolysis. Notably, the quantum yields were very low (Φ = 10^–3^ – 10^–4^ at 390 nm for Ni(II)–bpy aryl halides). 2) Temperature-dependent rate analysis revealed that there existed a modest barrier for excited-state Ni(II)–C(aryl) bond homolysis (∼4 kcal mol^–1^); this barrier was well below the predicted values for thermal dissociation from the ^3^d-d excited-state. 3) Quantum yields and rate constants for excited-state bond homolysis were highly wavelength-dependent; excitation into the lowest-energy MLCT (which relaxes to the ^3^d-d state) was unproductive. Only high energy light (>525 nm, ∼ 55 kcal mol^–1^) afforded aryl radicals. These experiments, alongside an expanded computational analysis, supported a dissociative MLCT/LMCT excited state for C(aryl)^•^ generation.^38^ Experiments further supported that Ni(I)–bpy halide species were the products of the unimolecular excited-state bond homolysis.^56^

In 2024, Hadt and co-workers expanded their photochemical analysis of Ni(II)–bpy aryl halides to a Ni(II)–^Ph^bpy chloride complex that features a covalent bond between the aryl ligand and the bpy backbone.^140^ This geometrically constrained complex demonstrated apparent photochemical stability over a broad wavelength range. However, evidence for Ni(I) generation upon irradiation was demonstrated through a reaction with an introduced aryl bromide, wherein substrate activation outcompeted radical recombination of the tethered aryl group. From transient absorption experiments, the structural constraint of the ligand prevented access to a ^3^d-d state by prohibiting the formation of a pseudo-*T*_d_ geometry (see Section 2 for electronic structure implications). The retention of light-promoted activation of an electrophile in this tethered complex again suggested that the triplet charge transfer dissociative pathway (MLCT/LMCT) likely facilitates excited-state Ni–C bond homolysis. As noted above, due to the small quantum yields for Ni(II)–C(aryl) homolysis, transient spectroscopies largely probe unproductive background excited-state relaxation processes that do not lead to the formation of Ni(I) intermediates and organic radicals, making the assignment of the photochemical pathway challenging and inconclusive.^41^

We briefly note that in addition to Ni(II)–C(aryl) bond homolysis, Ni(II)–C(alkyl) homolysis has been observed. For example, Park prepared Ni(II)–bpy methyl thiolate complexes to corroborate the possibility of carbon radical formation (see above).^109^ These complexes were designed to prefer irreversible Ni(II)–C(alkyl) bond homolysis and yielded ethane as the dominant photoproduct, thereby confirming methyl radical generation. Furthermore, aliphatic nickellacycles generated β-hydride elimination products under 390 nm irradiation. Similarly, Oderinde and co-workers questioned if Ni(II)–bpy dimethyl catalysts could mediate C(sp^2^)–C(sp^3^) cross-coupling reactions under visible light irradiation.^141^ Indeed, stoichiometric cross-coupled products were observed upon irradiation of the Ni(II) complex alongside 5-bromophthalide substrate using blue or violet light. Methyl radicals were confirmed via EPR radical-trap experiments followed by GC-MS analysis. These results reveal that alkyl radical formation through a Ni(II)–C(alkyl) bond homolysis pathway can be photoinduced with a variety of ligand backbones, including sulfur-ligated systems, an aromatic ligand (such as bpy), an aliphatic ligand (such as TMEDA), and occurs in both cyclic and acyclic compounds.

#### 3.2.2 Direct Excitation for Reductive Elimination

Excited states that serve to either 1) oxidize Ni via Ni-to-ligand change transfer transitions or 2) populate Ni-ligand antibonding orbitals via ligand field or ligand-to-Ni transitions have been suggested to promote direct intramolecular reductive elimination of organic substrates. In either case, experimental mechanistic analysis is lacking, marking an opportunity for interdisciplinary follow-up analysis.

Absorption of a photon by Ni(II)–bpy complexes has been suggested to drive excited state reductive elimination. Originally conceptualized by the McCusker and MacMillan groups in 2017,^96^ it was proposed that direct excitation of a Ni(II)–bpy aryl acetate complex ultimately resulted in the population of a low-energy triplet ligand field state, from which intramolecular reductive elimination could afford an aryl-acetate product with new C(sp^2^)–O bond and a reduced Ni(0)–bpy complex. Follow-up ns transient absorption on a mixture of Ir(III) photosensitizer and Ni(II)–bpy aryl acetate was conducted in 2020, wherein it was surmised that *Ir(III) underwent Dexter EnT to a ground-state Ni(II) complex (see Section 3.1.7 above), populating a long-lived triplet excited state. The nature of this state, i.e., charge transfer vs metal-centered, was not described. It was proposed, however, that this excited state was active for reductive elimination. Notably, ultrafast transient absorption on the independent excited-state dynamics of the Ni(II)–bpy aryl acetate was not presented. Computational assessment of the excited-state relaxation pathways of a Ni(II)–bpy aryl acetate was undertaken by Ma and co-workers that same year.^13^ Therein, it was proposed that direct excitation of the Ni(II) complex into a high energy, anti-Kasha^142,143^ Ni(III)–bpy^•–^ MLCT state was responsible for intramolecular reductive elimination, where the oxidation of the Ni(II) center serves as the driving force for substrate formation.^91,92,144^

Excited-state-driven reductive elimination was also seen by Lloyd-Jones and co-workers on Ni(II)–bpy aryl halides.^88^ Energy transfer from *Ir(III) to generate a triplet excited state Ni(II) complex resulted in the formation of an aryl halide substrate and Ni(0) – a reversible process as oxidative addition from Ni(0) is readily accessible at room temperature. Following these results, the Lin and Doyle groups noted that direct excitation of the Ni complex is also productive for the same reductive elimination/oxidative addition equilibrium.^110,145^ Indeed, this reversible light-driven chemistry was utilized for ligand exchange, promoting a (*retro*-)Finkelstein reaction. The long-lived excited state of Ni(II)–bpy aryl halides is a ^3^d-d state, which is populated after relaxation from higher-energy MLCT states.^37^ It is unclear if a charge transfer or metal-centered excited state is productive for reductive elimination; further experimental mechanistic work is still needed to elucidate the photophysics of this process.

Direct aryl-alkyl C(sp^2^)–C(sp^3^) reductive elimination from excited state, high-valent Ni(III/IV)–bpy complexes was demonstrated by Park in 2020.^108^ In this case, a LMCT promoted the cross-coupled product via the population of a Ni–C σ*-orbital, increasing the rate of substrate formation by up to a factor 10^5^ when compared to thermal, dark reactivity. Interestingly, these complexes were penta-coordinate, suggesting similarities to the light-driven Ni–X homolysis reactivity seen by Nocera, Mirica, and co-workers.^99,100,128^ A recent report by the Doyle group finds evidence for excited-state intramolecular C(sp^2^)–C(sp^3^) reductive elimination from Ni(II)–bpy aryl alkyl complexes;^110^ the mechanism for this process is presently unknown.

#### 3.2.3 Key Components of Direct Excitation

##### 3.2.3.1 Ni(II)–bpy Aryl Halides Are Light-Harvesting Species

The absorptive nature of Ni(II)–bpy aryl halide complexes is well described. The primary absorption features in the visible light region are Ni(II)-to-bpy MLCT in nature, with molar absorptivities in the range of 10^3^ – 10^4^ M^–1^ cm^–1^.^37,38^ Hadt and co-workers found that these transitions can be further separated into low- and high-energy MLCTs, with the various bpy π* acceptor orbitals marking the difference between the two.^12,38^ The low-energy bands are typically ∼415–580 nm, while the high-energy bands are found between ∼340–400 nm, with additional transitions extending into the higher energy region. If present, d-d bands are likely obscured by MLCT transitions due to their relatively low molar absorptivities (10^1^ – 10^2^ M^–1^ cm^–1^) (Figure 2). While low-temperature magnetic circular dichroism (MCD) can enhance ligand field transitions relative to charge transfer due to different selection rules relative to UV–vis, the greatest utility involves *C*-term intensity, which requires a paramagnetic ground state.^146^ The ground states of Ni(II)–bpy aryl halide compounds are diamagnetic, however, which would lead to an absence of *C*-term intensity. Thus, additional spectroscopic methods may be required to locate and assign ligand field excited states in these compounds. 2p3d resonant inelastic X-ray scattering (RIXS) may be a viable technique due to its ability to resonantly excite metal-based states.^147−149^ Higher energy incident wavelengths (<330 nm) populate ILCT (bpy π → π*) transitions. These have been found to relax into the MLCT manifold via transient absorption spectroscopy.^36,131^ Similarly, the MLCT transitions relax into d-d excited states before ultimately returning to the ground state.^37,150^

##### 3.2.3.2 Ni(I)–bpy Halide Is Produced via Direct Excitation, Not Ni(I)–bpy Aryl

Although the very low quantum yields for photoinduced Ni–ligand homolysis from Ni(II)–bpy aryl halides have made them challenging to study by transient spectroscopies, steady-state methods including UV–vis, NMR, EPR, and GC-MS have verified the formation of aryl radicals, not halogen radicals, upon light absorption (*vide supra*). Indeed, to the best of our knowledge, no direct experimental evidence of halogen radical production upon irradiation of Ni(II)–bpy aryl halide complexes has been provided. The exclusive observation of aryl radicals is in contrast to the early mechanistic proposals by Molander, Doyle, and Shields.^57,98^

These 2016 reports featuring key Ni(II/III)–X bond homolysis refer to the work by Nocera and co-workers developed in the context of HX splitting for solar energy storage.^99,100^ It is important to highlight the distinctions in the photohalogen elimination chemistry between these systems. In HX splitting, the Ni(III) species is an isolable, penta-coordinate Ni(III)–dppe trichloride complex (dppe = bis(diphenylphosphino)ethane). Here, a common dissociative excited-state surface is accessed upon 370 and 434 nm irradiation and was proposed to be responsible for the photoelimination of the apical chlorine ligand (Figure 10A). Based on time-dependent DFT calculations (TDDFT), the Ni(III)–Cl bond cleavage was attributed to a LMCT excitation into the unoccupied *p*(z)/*d*(z^2^) antibonding Ni-based hole, reducing the bond order between Ni(III) and the apical Cl to zero. The resulting photoproduct, Ni(II)(dppe)Cl_2_, exhibited a square-planar structure with a singlet ground state; no further halogen photoelimination occurred.

In 2022, Mirica and Na reported a study^128^ with a similar isolable, five-coordinate Ni(II) complex that featured a tridentate pyridinophane ligand (Figure 10B). Their proposed mechanism also featured chlorine photoelimination, both Ir(III)-facilitated via SET and under direct light excitation of the *S* = 1 Ni(II) complex. The latter displayed accessible triplet Ni-to-ligand charge transfer states. Excitation into one of these triplet states promoted an electron from the Ni 3*d*-orbital manifold, thus generating a transient Ni(III) complex. It was proposed that subsequent relaxation gave rise to a ^3^d-d state with significant σ* character along the Ni–Cl bond, triggering homolysis. Although chlorine radical trapping experiments were not presented, the lability of the Ni–Cl bonds was demonstrated in the chemically oxidized cationic Ni(III) complex.

Thus, photohalogen elimination is possible with penta-coordinate Ni(II/III) di- or trihalide complexes, but it is disfavored when using four-coordinate Ni(II/III) aryl halide complexes. The MLCT/LMCT process seen in Ni(II)–bpy aryl halide complexes is akin to that considered using TDDFT in the Ni(III)–Cl photohalogen elimination chemistry, but with the distinct difference that the LMCT originates from an aryl donor, not the halide. This is unsurprising, as the DFT predicted bond dissociation energy for Ni(II)–X homolysis is roughly twice that of Ni(II)–C(aryl).^37^ Interestingly, the Ni(II)–C(aryl) homolysis pathway is promiscuous with respect to the backbone ligand, as demonstrated by Park and co-workers and by Hadt and co-workers for aliphatic TMEDA ligands.^38,109^*It is therefore the presence of the aryl group that governs the selectivity for radical generation.*

This notion has been corroborated experimentally. Xue and co-workers^133^ demonstrated that even when replacing the halide with a stronger field ligand, as in the Ni(II)–bpy aryl cyanide complex, aryl radicals are still preferentially generated upon light absorption. Here, the starting Ni(II) complex was initially presented as a potential reactive intermediate in C(sp^2^)–N coupling reactions, with the authors suggesting that reductive elimination from a Ni(III) state would yield C(sp^2^)–N coupled products. Reductive elimination was indeed observed after single-electron oxidation via an excited photosensitizer. However, in the absence of photosensitizer, no reductive elimination occurred; the reaction instead resulted in biphenyl product formation, suggesting photochemical Ni(II)–C(aryl) bond homolysis (which was later confirmed by EPR). The formation of the d^9^ Ni(I)–bpy cyanide intermediate upon irradiation of the parent Ni(II) structure was also confirmed by EPR.^133^ Furthermore, recent work suggests that high-spin (*S* = 1) Ni(II)–bpy dihalide (and Ni(II)–TMEDA dihalide) engage in photohalogen elimination upon direct excitation with high-energy light, thereby recovering the photohalogen elimination pathway by removing the aryl group from the parent Ni(II) complex.^110,151−153^ Similarly, photopseudo-halogen elimination from Ni(II)–bpy diacetate has been proposed by Xue and co-workers when using purple light.^154−156^

##### 3.2.3.3 Ni(I)–bpy Halide Is the Active Species for Oxidative Addition, but Also Suffers from off-Cycle Dimerization

The potency of Ni(I)–bpy halide for the oxidative addition of aryl halide substrates has been demonstrated experimentally. Hammett analysis by the groups of Bird and MacMillan, Sigman, and Doyle suggested that aryl iodides and bromides are activated via a concerted, two-electron oxidative addition mechanism.^60,82,157^ When studying reactivity of Ni(I)–bpy halides with aryl chlorides, Hadt and co-workers found a relatively higher ρ value from Hammett analysis, which suggested the activation step is characterized by a concerted, two-electron nucleophilic substitution mechanism (*S*_N_Ar).^56^ For this, the nucleophilic site is the doubly occupied 3*d*(z^2^) orbital, which carries out a two-electron transfer into the C(sp^2^)–Cl σ* orbital. It was found that the reactivity of the Ni(I)–bpy halide species can be tuned via the energy of this orbital; bpy ligand modifications alter the effective nuclear charge (Z_eff_) of the Ni(I) and serve to increase (via electron-donating groups) or decrease (via electron-withdrawing groups) the reactivity of the complex toward substrate activation.

Although one might speculate that cross-coupling reactions would be accelerated by increased Ni(I) concentration in solution, Nocera and co-workers found that doing so (by increasing the photon flux of the irradiation source) was actually detrimental to productive catalysis.^95^ At a high concentration of Ni(I), the low-valent species is prone to either direct dimerization to yield formal [Ni(I)–bpy halide]_2_ complexes (Figure 11) or to aggregation with the parent Ni(II)–bpy aryl halide species–both of which are off-cycle products. On the basis of X-ray photoelectron spectroscopy on a synthesized [Ni(I)–bpy halide]_2_ complexes, Hazari and co-workers described this species as a dimeric Ni(II)–bpy^•–^ halide.^129^ It was also found to be inert toward aryl halide substrates.^129,157^ Importantly, the same electronic structure effects that govern the reactivity of Ni(I)–bpy halide species also dictate their tendency toward this dimerization pathway; electron-rich ligands promote dimerization, while electron-deficient ligands slow or fully inhibit room-temperature dimerization (Δ*G*^‡^ ∼ 25 kcal mol^–1^).^56^*Therefore, the relative rates of Ni(I)–bpy halide formation, substrate activation, and off-cycle dimerization should be considered when optimizing catalytic cycles.*

##### 3.2.3.4 Penta-Coordinate Ni(III) Undergoes Reductive Elimination and/or Comproportionation to Close the Cycle

As has been discussed above, Ni(III) is prone to rapid reductive elimination. Indeed, mechanistic work by Mirica and Doyle and co-workers found that reductive elimination is facile, even at low temperatures.^110,128^ In good agreement with these experimental observations, DFT calculations by Hadt and co-workers suggest that reductive elimination of an aryl halide from a Ni(III)(bpy)(aryl)X_2_ species is effectively barrierless.^56^ Nonetheless, under continuous irradiation and formation of both Ni(I) and Ni(III) species, there exists a non-negligible resting state concentration of Ni(III) in solution that can undergo comproportionation with Ni(I) to afford *S* = 0 Ni(II)–bpy aryl halide and *S* = 1 Ni(II)–bpy dihalide.^158,159^ This has been experimentally demonstrated by Doyle and co-workers in 2022^84^ and 2024^110^ and by Hadt and co-workers in 2023^56^ by NMR analysis. The low-spin Ni(II)–bpy aryl halide is thereby returned to the catalytic cycle, where it can absorb a photon and be transformed anew to Ni(I)–bpy halide (Figure 11). However, *S* = 1 Ni(II)–bpy dihalide accumulates following comproportionation over multiple turnovers, potentially diverting the cycle to one beginning at the high spin species. Detailed mechanistic work on the photophysical pathway upon direct excitation of Ni(II)–bpy dihalide complexes is still needed.

## 4 Conclusions and Outlook

In this Review, we have provided a summary of the various mechanisms presented for metallaphotoredox reactions featuring Ni(II)–bpy catalysts. A few common mechanistic observations have arisen:

1) Commonly employed photosensitizers such as cyclometalated Ir(III) heteroleptic complexes can engage in numerous excited-state quenching pathways, including ^3^EnT and reductive/oxidative SET, complicating mechanistic analysis. Furthermore, in metallaphotoredox cycles there are often numerous species capable of quenching the photosensitizer excited state, organic and inorganic alike. The precise mechanism of quenching, and the quenching species, are still largely debated. Detailed experimental mechanistic work (including Stern–Volmer analysis) is needed for individual steps in Ir/Ni dual photocatalytic cycles.
2) The electronic structure of the Ni(II)–bpy species present in the reaction governs the specific mechanistic pathway it follows; this electronic structure is highly sensitive to both ligands and the surrounding environment. To highlight a few key structures, Ni(II)–bpy aryl halides and Ni(II)–bpy aryl acetates are both photosensitized species and direct light-harvesters. Ni(II)–bpy aryl halides undergo photochemical Ni(II)–C(aryl) bond homolysis, a key step for initiation into Ni(I)/Ni(III) dark cycles, while Ni(II)–bpy aryl acetates have been proposed to follow an excited-state intramolecular reductive elimination path. Ni(II)–bpy dihalide species are primarily photosensitized complexes, typically resulting in a reduced Ni(I)–bpy halide. However, with high-energy light, these complexes have been proposed to also engage in direct *Ni(II)–X bond homolysis to form the same Ni(I) intermediate.
3) Regardless of the Ni(I)–bpy halide photochemical generation mechanism, a consistent Ni(I)/Ni(III) cycle has been proposed for most cross-coupling reactions. The key catalytic steps consist of an oxidative addition of electrophile (such as aryl halide) to Ni(I) to form a reactive Ni(III)(bpy)(Ar)X_2_ intermediate. This Ni(III) can undergo X ligand exchange with a nucleophile (presumably facilitated by a base), followed by reductive elimination to form the cross-coupled product and regenerate Ni(I)–bpy halide. Continuous irradiation is often necessary, likely due to the formation of off-cycle, catalytically inactive Ni(II)–bpy dihalide via Ni(I)/Ni(III) comproportionation. However, continuous high photon flux leads to an accumulation of Ni(I)–bpy halide, resulting in aggregation with other Ni species in solution or dimerization to [Ni(I)/Ni(I)] off-cycle products.

Viewing these light-driven mechanisms from an electronic structure perspective has granted key considerations for the steps in each cycle, particularly pertaining to Ni-based intermediates. Although tremendous advancements have been made in reaction development in this field, unified, experimentally supported mechanisms are still lacking for many of the reactions. Due to the inherent complexity in these cycles, care should be taken to evaluate individual steps independently. We hope that presenting this Review from the standpoint of the outlined key considerations has provided a model logic from which to conduct such analyses. Finally, thermally driven enantioselective cross-coupling catalysis, often utilizing metal-based reductants instead of photochemical processes, feature similar complementary mechanistic considerations.^6,10,61,62,160,161^ As such, mechanistic studies in this field are of direct relevance for designing future studies. We hope research groups from the physical, organic, and inorganic fields will take on interdisciplinary research to elucidate the precise mechanisms of Ni-mediated cross-coupling catalysis, providing rationale to improve product scope and reaction efficiency and laying a foundation to extend catalyst activity to other transition metals.
